# Orchestrated activation of mGluR5 and CB_1_ promotes neuroprotection

**DOI:** 10.1186/s13041-016-0259-6

**Published:** 2016-08-20

**Authors:** Edleusa M. L. Batista, Juliana G. Doria, Talita H. Ferreira-Vieira, Juliana Alves-Silva, Stephen S. G. Ferguson, Fabricio A. Moreira, Fabiola M. Ribeiro

**Affiliations:** 1 Departamento de Bioquimica e Imunologia, Instituto de Ciencias Biologicas, Universidade Federal de Minas Gerais, Ave. Antonio Carlos 6627, Belo Horizonte, MG CEP: 31270-901 Brazil; 2 Department of Cellular and Molecular Medicine, Faculty of Medicine, University of Ottawa, Ottawa, K1H8M5 Canada; 3 Departamento de Farmacologia, Instituto de Ciencias Biologicas, Universidade Federal de Minas Gerais, Belo Horizonte, 31270-901 Brazil

**Keywords:** mGluR5, CB1, Cell death, AKT, ERK1/2

## Abstract

The metabotropic glutamate receptor 5 (mGluR5) and the cannabinoid receptor 1 (CB_1_) exhibit a functional interaction, as CB_1_ regulates pre-synaptic glutamate release and mGluR5 activation increases endocannabinoid synthesis at the post-synaptic site. Since both mGluR5 and CB_1_ promote neuroprotection, we delineated experiments to investigate a possible link between CB_1_ and mGluR5 activation in the induction of neuroprotection using primary cultured corticostriatal neurons. We find that either the pharmacological blockade or the genetic ablation of either mGluR5 or CB_1_ can abrogate both CB_1_- and mGluR5-mediated neuroprotection against glutamate insult. Interestingly, decreased glutamate release and diminished intracellular Ca^2+^ do not appear to play a role in CB_1_ and mGluR5-mediated neuroprotection. Rather, these two receptors work cooperatively to trigger the activation of cell signaling pathways to promote neuronal survival, which involves MEK/ERK1/2 and PI3K/AKT activation. Interestingly, although mGluR5 activation protects postsynaptic terminals and CB_1_ the presynaptic site, intact signaling of both receptors is required to effectively promote neuronal survival. In conclusion, mGluR5 and CB_1_ act in concert to activate neuroprotective cell signaling pathways and promote neuronal survival.

**Electronic supplementary material:**

The online version of this article (doi:10.1186/s13041-016-0259-6) contains supplementary material, which is available to authorized users.

## Introduction

The metabotropic glutamate receptor 5 (mGluR5) is a G-protein coupled receptor (GPCR) that is present at the postsynaptic site and is involved in a wide variety of processes, including motor behaviour, nociception, memory and neurodegeneration [1, 10, 12, 18, 22, 53]. mGluR5 stimulation promotes activation of Gα_q/11_ proteins, which trigger the activation of phospholipase Cβ1 (PLCβ1), leading to diacylghycerol (DAG) and inositol-1,4,5-triphosphate (IP_3_) formation and the release of Ca^2+^ from intracellular stores. In addition, most likely by coupling to homer proteins, mGluR5 also activates other cell signaling pathways, including extracellular-signal-regulated kinase 1/2 (ERK1/2) and AKT [7, 10, 21, 35, 48, 50]. We have recently demonstrated that mGluR5 positive allosteric modulators (PAMs) preferentially activate neuroprotective cell signaling pathways and prevent neuronal cell death in primary cultured striatal neurons, as well as in a mouse model of Huntington’s disease (HD) [10, 11]. Importantly, the memory loss observed in this mouse model of HD can be rescued by the treatment with the mGluR5 PAM, CDPPB [10].

Activation of the endocannabinoid system also mediates neuroprotection [2, 36, 52]. Δ^9^-tetrahydrocannabinol (Δ^9^-THC), the main active component of the plant *Cannabis sativa*, as well as endocannabinoids, including anandamide and 2-arachidonoylglycerol (2-AG), activate cannabinoid receptor 1 (CB_1_), which is a Gα_i/o_–coupled receptor mainly found at the presynaptic terminal. Endocannabinoids are synthesized on demand at the post-synaptic terminal and act on cannabinoid receptors to prevent the release of neurotransmitters, including gamma-aminobutyric acid (GABA) and glutamate [26, 44, 66]. Therefore, CB_1_ can diminish excitotoxic neurotransmission by lessening pre-synaptic glutamate release [28, 36]. Similarly to mGluR5, CB_1_ activation stimulates neuroprotective ERK1/2 and AKT cell signaling pathways, which are involved in the control of cell survival [4, 15, 63, 65].

Previous studies have demonstrated that mGluR5 and CB_1_ exhibit a functional interaction that is implicated in a wide variety of processes, including pain [47], cocaine addiction [13], Fragile X syndrome [57], anxiety and memory [62]. Interestingly, activation of mGluR5 stimulates endocannabinoid synthesis by increasing intracellular Ca^2+^ [6]. However, mGluR5 can also increase 2-AG synthesis in a mechanism that is independent of Ca^2+^ [45]. Homer proteins, which bind to mGluR5, also bind to the two key proteins responsible for 2-AG synthesis, PLCβ, which is activated by mGluR5, and diacylglycerol lipase-α (DGL-α) [23, 25]. The formation of this protein complex enables the rapid formation of 2-AG following mGluR5 stimulation. At the same time, by activating pre-synaptic CB_1_, 2AG blunts glutamate release and as a consequence mGluR5 activation [36, 52]. However, it is still not known whether these two receptors could function cooperatively to promote neuroprotection. To address that, we have employed pharmacological and genetic manipulations to access neuronal cell death in primary cultured corticostriatal neurons under glutamate insult. We find that either the pharmacological blockade or genetic ablation of either mGluR5 or CB_1_ abolishes both CB_1_- and mGluR5-mediated neuroprotection. Moreover, these two receptors trigger the same cell signaling pathways to promote neuroprotection, which involves activation of ERK1/2 and AKT.

## Methods

### Materials

Neurobasal medium, N2 and B27 supplements, GlutaMAX (50.0 mg/ml penicillin and 50 mg/ml streptomycin), Live/Dead viability assay, anti-rabbit Alexa Fluor 488 antibody, anti-mouse Alexa Fluor 546, DAPI (4′,6-Diamidino-2-Phenylindole, Dihydrochloride) and anti-PSD95 were purchased from Thermo Fisher Scientific. 3-Cyano-*N*-(1,3-diphenyl-1*H*-pyrazol-5-yl)benzamide (CDPPB), 2-methyl-6-(phenylethynyl)-pyridine (MPEP), 2-(2-Amino-3-methoxyphenyl)-4H-1-benzopyran-4-one (PD98059), 2-(4-Morpholinyl)-8-phenyl-1(4H)-benzopyran-4-one hydrochloride (LY294002), N-arachidonoylethanolamine (anandamide), 2-Arachidonoylglycerol (2-AG) and arachidonyl-2′-chloroethylamide (ACEA) were purchased from Tocris Cookson Inc. Cyclohexylcarbamic acid 3′-carbamoyl-biphenyl-3-yl ester (URB597), 4-[Bis(1,3-benzodioxol-5-yl)-hydroxymethyl]piperidine-1-carboxylate 4-nitrophenyl ester (JZL184) and 1-(2,4-Dichlorophenyl)-5-(4-iodophenyl)-4-methyl-N-1-piperidinyl-1H-pyrazole-3-carboxamide (AM251) were purchased from Cayman Chemical. Horseradish peroxidase-conjugated anti-rabbit IgG secondary antibody was from BioRad. Western Blotting ECL Prime detection reagents were from GE Healthcare and Immobilon Western Chemiluminescent HRP Substrate was from Millipore. Anti-phospho AKT and anti-AKT rabbit monospecific clonal antibodies were from DB Biotech. Anti-phospho ERK1/2 (thr202/Tyr204) and anti-ERK1/2 rabbit antibodies were from Cell Signaling (USA). Anti-CB_1_ (1–77) rabbit pAB antibody was from Calbiochem. Anti-syantaxin 1A was from Santa Cruz. All other biochemical reagents were purchased from Sigma–Aldrich.

### Mouse model

C57/BL6 mice (25–30 g) were purchased from the animal facility (CEBIO) located at the Universidade Federal de Minas Gerais (UFMG). mGluR5^−/−^ mice B6;129-Grm5^tm1Rod^/J (*mGluR5*^*−/−*^) [34] were purchased from Jackson Laboratory (Bar Harbor, ME) and PI3Kγ^−/−^ mice were kindly provided by Dr. M. M. Teixeira [51]. Mice were housed in an animal care facility at 23 °C on a 12 h light/12 h dark cycle with food and water provided ad libitum. Animal care was in accordance with the Universidade Federal de Minas Gerais Ethics Committee on Animal Experimentation (CETEA) and all Animal procedures were approved by CETEA/UFMG, protocol number 3/2011.

### Neuronal primary culture preparation

Neuronal cultures were prepared from the striatal and cortical regions of E15 WT, mGluR5^−/−^ or PI3Kγ^−/−^ embryo brains. After dissection, corticostriatal tissue was submitted to trypsin digestion followed by cell dissociation using a fire-polished Pasteur pipette. Cells were plated on poly-L-ornithine coated dishes in Neurobasal medium supplemented with N2 and B27 supplements, 2 mM GlutaMAX, 50 μg/ml penicillin and 50 μg/ml streptomycin. Cells were incubated at 37 °C and 5 % CO_2_ in a humidified incubator and cultured for 10 to 12 days in vitro (DIV) with medium replenishment every 4 days.

### siRNA electroporation

Neurons were submitted to electroporation during preparation of primary cultures using mouse neuron Nucleofector kit (Lonza), according to manufacture instructions. Before being plated on coated dishes, a total of 2 × 10^6^ corticostriatal neurons were incubated with the electroporation reagent and 30 pmol of either NC- or CB_1_-siRNA (Santa Cruz). Electroporation was performed using an AMAXA nucleofector II Device apparatus and program 0–005. Neurons were then transferred to poly-L-ornithine coated dishes in supplemented Neurobasal medium. Media was replaced 4 h later and, following that, cells were incubated in the same conditions as not electroporated neurons.

### Neuronal stimulation

To determine the appropriate drug concentration to be used, concentration-response experiments were performed for each drug used (data not shown), unless concentration-response curves have already being performed in previous publications from our group [10, 63]. Neuronal primary cultures obtained from WT, mGluR5^−/−^ or PI3Kγ^−/−^ embryos were incubated with either vehicle (Hank’s balanced salt solution: HBSS) or 50 μM glutamate, in the presence or absence of 100 nM CDPPB, 1 nM URB597, 10 nM JZL184, 10 nM anandamide, 10 nM 2-AG, 1 nM ACEA for 4 h at 37 °C, as indicated in the *Figure Legend*. When 1 μM MPEP, 10 nM AM251, 10 μM PD98059 or 1 μM LY294002 was used, it was added 5 min prior to and kept during the rest of the incubation. Following this incubation, neuronal cultures were used for cell death assays, glutamate release experiments, [Ca^2+^]i measurements, ERK1/2 and AKT activation assays or immunofluorescence labeling experiments.

### Cell death assay

Neurons were stimulated with drugs, as indicated in the *Figure Legend*, and cell death was determined by Live/Dead viability assay, as described previously [11]. Briefly, neurons were stained with 2 μM calcein acetoxymethyl ester (AM) and 2 μM ethidium homodimer-1 for 15 min and the fractions of live (calcein AM positive) and dead (ethidium homodimer-1 positive) cells were determined. Neurons were visualized by fluorescence microscopy FLoid® Cell Imaging Station (Thermo Fisher Scientific) and scored by a blinded observer. A minimum of 150 cells were analyzed per well in triplicate using ImageJ software. Dead cells were expressed as a percentage of the total number of cells.

### Glutamate release experiment

Glutamate released by primary cultured neurons was measured indirectly by the fluorescence increase due to the production of NADPH in the presence of glutamate dehydrogenase type II and NADP^+^ [42]. Neuronal cultures seeded on 96-well plates were incubated with CaCl_2_ (1 mM) and NADP^+^ in HBSS and analyzed in a spectrofluorometer (Synergy 2, BioTek® Instruments, Inc.). Five min later, glutamate dehydrogenase (50 units per well) was added and reading was restarted until the fluorescence reached balance (approximately 5 min). After that, neuronal cultures were stimulated for 5 min with drugs as described in the *Figure Legend*. Calibration curves were done in parallel by adding known amounts of glutamate (5 nM/μl) to the reaction medium. The experimental data were expressed as percentage, taking glutamate released by neurons stimulated with 50 μM glutamate as 100 %. The experiments were performed at 37 °C, in duplicate well for each condition, with excitation wavelength of 360 nm and emission of 450 nm.

### Measurement of intracellular Ca^2+^ concentration

Neuronal cultures seeded on 96-well plates were loaded with 0.2 μM Fura-2 AM for 20 min at 37 °C and stimulated with drugs, as described in the *Figure Legend*. Neurons were washed with HBSS and illuminated with alternating 340- and 380-nm light, with the 510-nm emission detected using a spectrofluorometer. At the end of each experiment sodium dodecyl sulfate (SDS) 10 % (0.1 % final) was added to obtain *R*_max_ followed by 3 M Tris + 400 mM EGTA (pH 8.6) for *R*_min_, as described by [16]. The increase in [Ca^2+^]_i_ promoted by 50 μM glutamate was taken as 100 %. All experiments were performed in triplicate wells for each condition.

### Immunoblotting

Following drug stimulation, neurons were lysed in RIPA buffer (0.15 M NaCl, 0.05 M Tris-HCl, pH 7.2, 0.05 M EDTA, 1 % nonidet P40, 1 % Triton X-100, 0.5 % sodium deoxycholate, 0.1 % SDS) containing protease inhibitors (1 mM AEBSF and 10 μg/ml of both leupeptin and aprotinin). 100 μg of total cellular protein for each sample was subjected to SDS-PAGE, followed by electroblotting onto nitrocellulose membranes. Membranes were blocked with 5 % BSA in wash buffer (150 mM NaCl, 10 mM Tris–HCl, pH 7.4, and 0.05 % Tween 20) for 1 h and then incubated with either rabbit anti-phospho AKT (S473) (1:1000) or rabbit anti-phospho ERK1/2 (Thr202/Thr204) (1:1000) antibodies in wash buffer containing 3 % BSA for 2 h at room temperature. Membranes were rinsed three times with wash buffer and then incubated with secondary peroxidase-conjugated anti-rabbit IgG antibody diluted 1:5000 in wash buffer containing 3 % skim milk for 1 h. Membranes were rinsed three times with wash buffer, incubated with ECL prime western blotting detection reagents and scanned and analysed by ImageQuant LAS 4000 (GE Healthcare). Antibodies were then stripped and membranes were incubated with either rabbit anti-AKT (1:1000) or rabbit anti-ERK1/2 (1:1000) antibodies for 2 h and probed with secondary antibody anti-rabbit IgG 1:5000 to determine total AKT and ERK1/2 expression. Non-saturated, immunoreactive AKT and ERK1/2 bands were quantified by scanning densitometry. Immuno-band intensity was calculated using ImageJ software and the number of pixels of AKT and ERK1/2 phospho-bands was divided by the number of pixels of total AKT and ERK1/2 to normalize phosphorylation levels of kinases to total kinase expression. In the case of CB_1_ protein expression assessments, membranes were blocked with 5 % skim milk and 5 % BSA in wash buffer (150 mM NaCl, 10 mM Tris-HCl, pH 7.4, and 0.1 % Tween 20) for 24 h and then incubated with either rabbit anti-CB_1_ (1:1000) or rabbit anti-actin (1:1000) antibodies in wash buffer containing 3 % skim milk overnight at 4 °C. Membranes were rinsed three times with wash buffer and then incubated with secondary peroxidase-conjugated anti-rabbit IgG antibody diluted 1:5000 in wash buffer containing 3 % skim milk for 1 h. Membranes were rinsed three times with wash buffer, incubated with ECL western blotting detection reagents and scanned and analysed by ImageQuant LAS 4000.

### Immunofluorescence and imaging

Corticostriatal neurons were stimulated, as describe in the *Figure Legend*, washed twice in PBS and fixed with 4 % formaldehyde in PBS for 20 min. After fixation, cells were washed with PBS and preincubated with a permeabilization solution (PBS, 0.2 % Triton, and 1 % bovine serum albumin) for 20 min. Following that, mouse anti-syntaxin 1A (1:500) or rabbit anti-PSD95 (1:500) antibodies were added to cells and incubated in permeabilization solution for 16 h at 4 ° C. Cells were washed and incubated with goat anti-mouse and anti-rabbit antibodies conjugated to Alexa Fluor 546 1:500 and Alexa Fluor 488 1:500, respectively, for 60 min in permeabilization solution. Following that, cells were washed and stained with DAPI 1:1000 for 10 min. Fluorescence microscopy was performed using a Zeiss LSM 880 confocal system equipped with a 40x/1.30 oil DIC M27 objective. Image acquisition was done by a blind observer using the Zen 2 software. Settings for wavelength detection of immunolabeled proteins were adjusted as follows: DAPI was imaged by detection between 410 and 496 nm, Alexa Fluor 488-labeled anti-PSD95 antibody was detected between 499 and 552 nm, and Alexa Fluor 546-labeled anti-syntaxin 1A antibody was detected between 552 and 679 nm. Sequential excitation of fluorophores was performed using 405, 488, 532 nm lasers for DAPI, Alexa Fluor 488 and Alexa Fluor 546, respectively. Fluorescence intensity of pixel grey levels was obtained using ImageJ software and total intensity was normalized by the total number of cells (DAPI-labeled nucleus).

### Data analysis

Means ± SEM are shown for the number of independent experiments indicated in *Figure Legends*. GraphPad Prism™ software was used to analyze data for statistical significance. Statistical significance (*p* <0.05) was determined by analysis of variance (ANOVA) testing followed by Bonferroni post-hoc Multiple Comparison Testing.

## Results

### The neuroprotection induced by CDPPB, URB597 and JZL184 can be blocked by both CB_1_ and mGluR5 antagonists

**Fig. 1:**
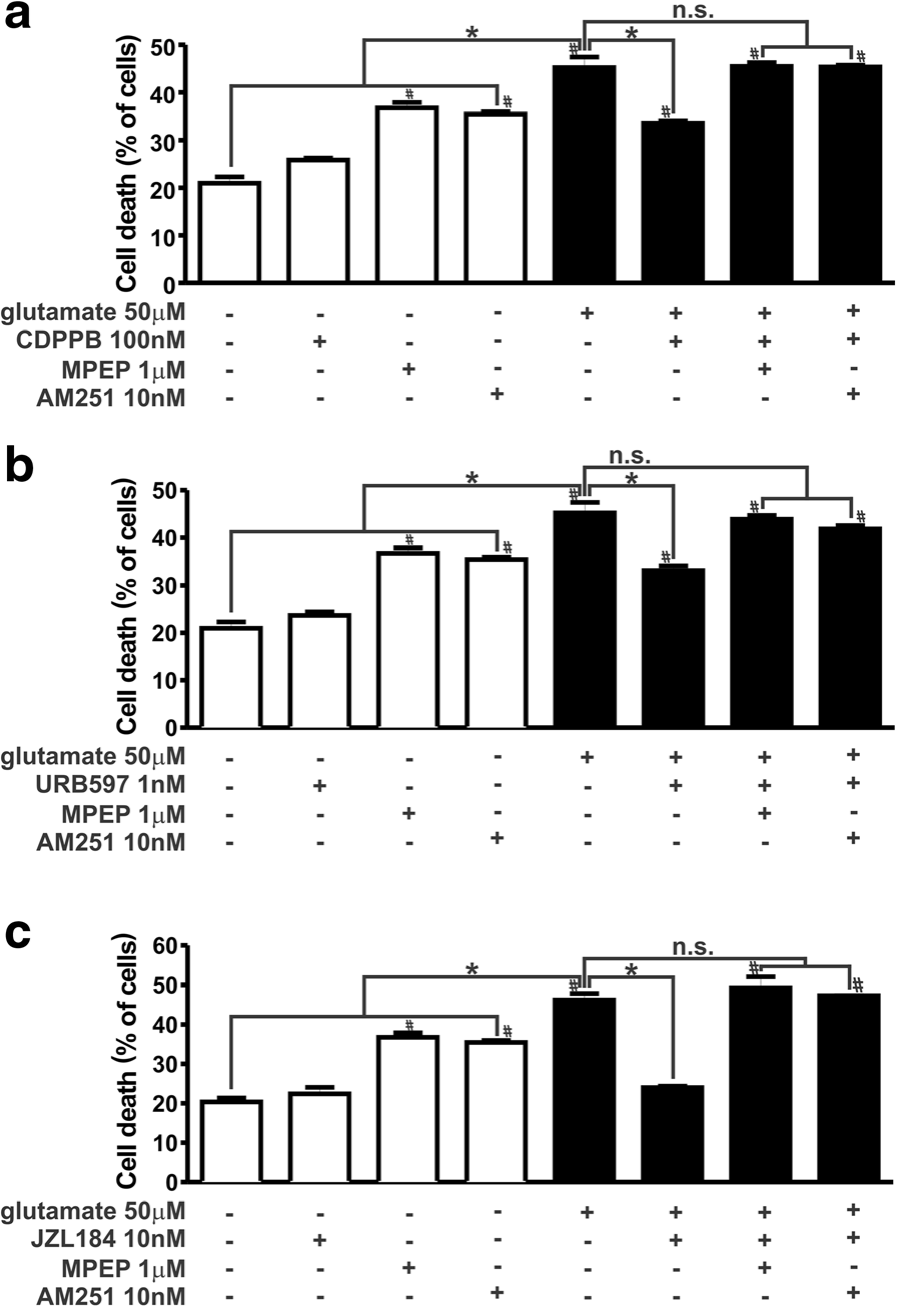
The neuroprotection induced by CDPPB, URB597 and JZL184 can be blocked by both CB_1_ and mGluR5 antagonists. Graphs show cell death levels of primary cultured corticostriatal neurons that were either untreated (−) or treated (+) with 50 μM glutamate, 1 μM MPEP, 10 nM AM251, 100 nM CDPPB (**a**), 1 nM URB597 (**b**) and 10 nM JZL184 (**c**) for 4 h. Data represent the means ± SEM of four independent experiments. n.s. indicates not significant, * indicates significant difference as compared to glutamate treated neurons (*p* <0.05) and # indicates significant difference as compared to untreated neurons (*p* <0.05)

We have previously demonstrated that CDPPB, an mGluR5 PAM, promotes survival of primary cultured striatal neurons and rescues the neuronal cell loss observed in a mouse model of HD [10, 11]. In order to investigate whether CB_1_ could be involved in mGluR5-mediated neuroprotection, we prepared primary neuronal cultures from the cortex and striatum, which contain mostly GABAergic and glutamatergic neurons [11, 40]. Incubation of these cultures with 50 μM glutamate for 4 h promoted the death of 45–50 % of the cells (Fig. 1). Next, we performed concentration-response experiments to determine the concentrations of MPEP and AM251 that were effective to block mGluR5 and CB_1_, respectively, without leading to high levels of neuronal death. We found that 1 μM MPEP and 10 nM AM251 were the lowest concentrations of antagonists that were effective to block the receptors (data not shown). Although the levels of neuronal cell death triggered by both antagonists were higher than basal levels, they were not as high as glutamate-induced neuronal cell death (Fig. 1). Thus, 1 μM MPEP and 10 nM AM251 were the concentrations used in this study.

In agreement with previously published data from our group, 100 nM CDPPB prevented glutamate-induced neuronal cell death and the mGluR5 antagonist MPEP (1 μM) abrogated CDPPB-induced neuroprotection (F_7,32_ = 72.66, *p* <0.0001; Fig. 1a). To check whether CB_1_ was involved in CDPPB-mediated neuroprotection, neurons were pre-treated with the CB_1_ antagonist, AM251. Interestingly, 10 nM AM251 efficiently abolished CDPPB-induced neuroprotection (Fig. 1a), indicating that CB_1_ was involved in mGluR5-mediated neuroprotection.

There are many pharmacological tools to manipulate the cannabinoid system. Anandamide and 2-AG are direct agonists of cannabinoid receptors. However, these compounds are very rapidly degraded by the enzymes fatty acid amide hydrolase (FAAH) and monoacylglycerol lipase (MGL), respectively [9, 20, 60]. On the other hand, the degrading enzymes FAAH and MGL can be inhibited by URB597 and JZL184, respectively, increasing the levels of endocannabinoids [27, 33]. To test whether increased levels of anandamide and 2-AG could rescue cell death promoted by 50 μM glutamate, corticostriatal neurons were treated with either 1 nM URB597 or 10 nM JZL184. Both URB597 (F_7,32_ = 58.67, *p* <0.0001; Fig. 1b) and JZL184 (F_7,24_ = 60.18, *p* <0.0001; Fig. 1c) were efficient to promote neuroprotection under these conditions. Induction of neuroprotection was dependent on CB_1_, as AM251 blocked both URB597- (Fig. 1b) and JZL184-induced (Fig. 1c) neuronal survival. However, MPEP was also capable of abrogating URB597- (Fig. 1b) and JZL184-induced (Fig. 1c) neuroprotection. mGluR5 was previously shown to be important for endocannabinoid synthesis and its blockade can diminish endogenous levels of cannabinoids [23, 25]. Thus, we hypothesized that when mGluR5 was blocked, inhibition of endocannabinoid degradation would not increase cannabinoids to levels high enough to promote neuroprotection. In this case, CB_1_ direct agonist would be able to promote neuroprotection even if mGluR5 was blocked. To test this hypothesis, we used cannabinoid receptors direct agonists, including anandamide and 2-AG, as well as ACEA, a CB_1_ specific agonist, to rescue glutamate-induced neuronal cell death. The three tested cannabinoid receptor direct agonists were capable of rescuing glutamate-induced neuronal cell death and AM251 was efficient to block this effect (F_13,70_ = 76.98, *p* <0.0001; Additional file 1: Figure S1). However, mGluR5 blockade by MPEP only partially abolished anandamide-, 2-AG- and ACEA-induced neuronal survival (Additional file 1: Figure S1). Thus, mGluR5 activity was not as important when CB_1_ direct agonists were used, as there was no need for endocannabinoid synthesis.

### CDPPB, URB597 and JZL184 treatment activate ERK1/2 and AKT

**Fig. 2:**
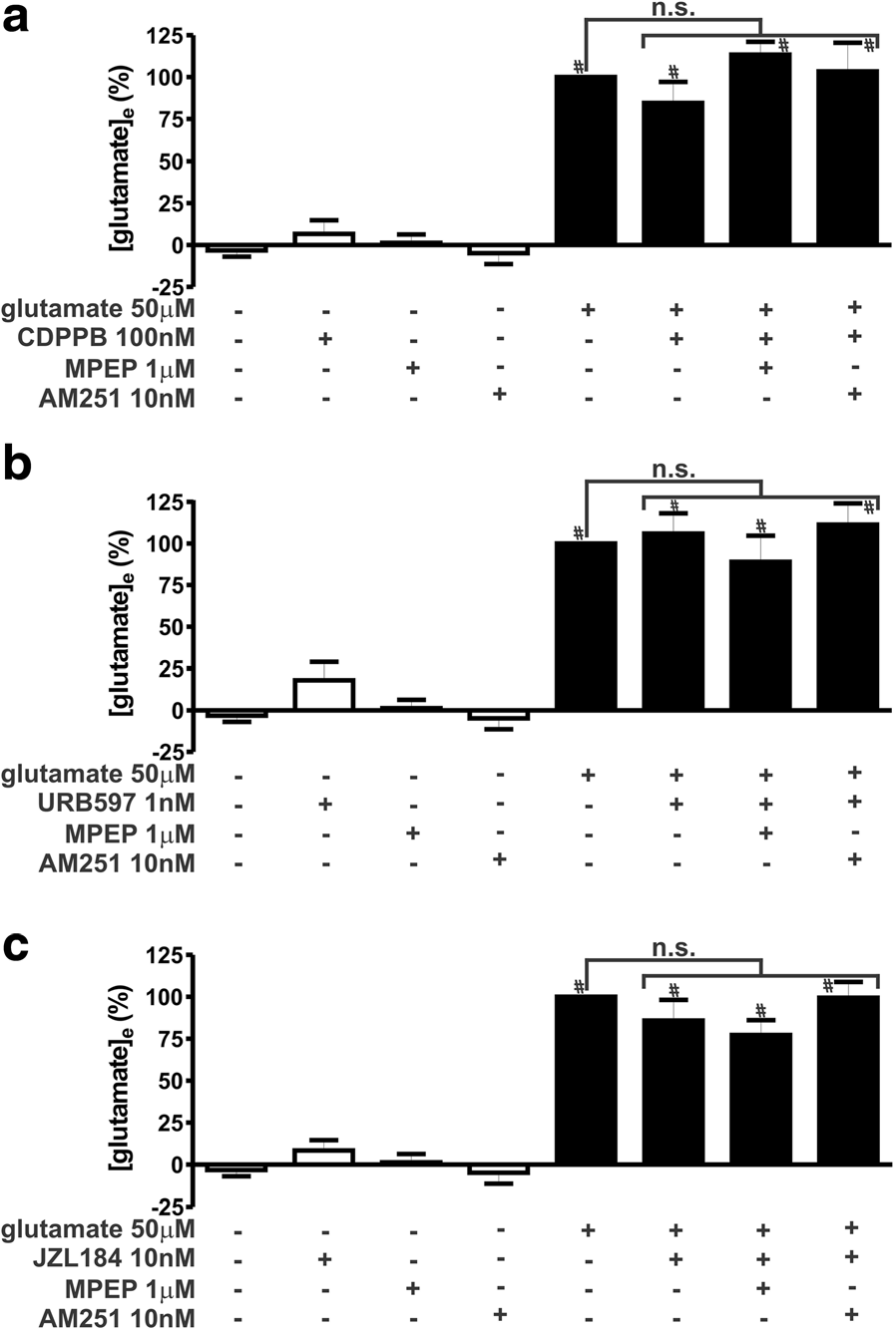
CDPPB, URB597 and JZL184 treatment do not alter glutamate extracellular concentration. Graphs show extracellular glutamate concentration ([glutamate]_e_) levels in primary cultured corticostriatal neurons that were either untreated (−) or treated (+) with 50 μM glutamate, 1 μM MPEP, 10 nM AM251, 100 nM CDPPB (**a**), 1 nM URB597 (**b**) and 10 nM JZL184 (**c**) for 5 min. Data represent the means ± SEM of four independent experiments. n.s. indicates not significant and # indicates significant difference as compared to untreated neurons (*p* <0.05)

**Fig. 3:**
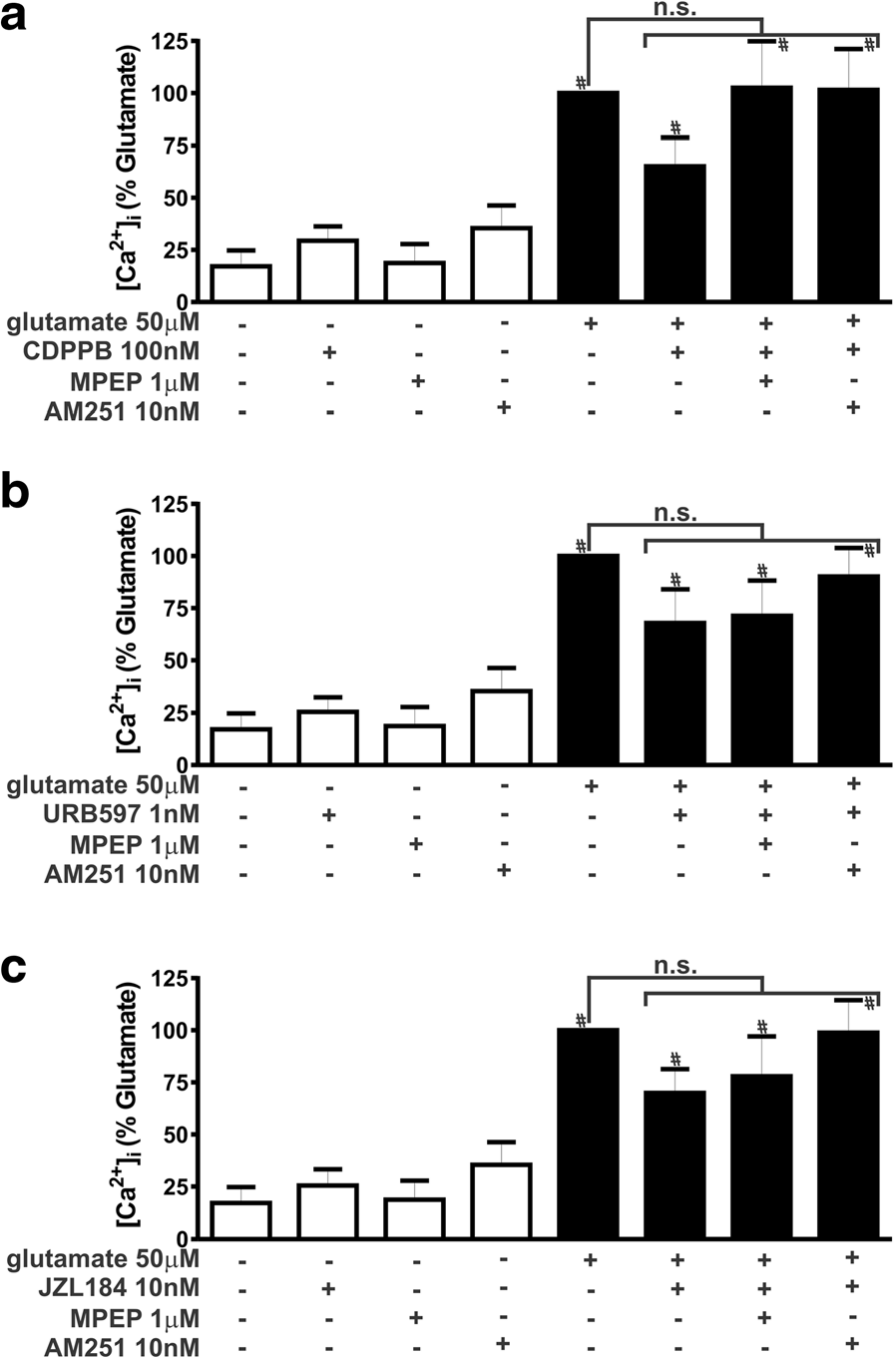
CDPPB, URB597 and JZL184 treatment do not modify intracellular Ca^2+^ concentration. Graphs show intracellular Ca^2+^ concentration ([Ca^2+^]_i_) levels in primary cultured corticostriatal neurons that were either untreated (−) or treated (+) with 50 μM glutamate, 1 μM MPEP, 10 nM AM251, 100 nM CDPPB (**a**), 1 nM URB597 (**b**) and 10 nM JZL184 (**c**) for 5 min. Data represent the means ± SEM of seven independent experiments. n.s. indicates not significant and # indicates significant difference as compared to untreated neurons (*p* <0.05)

Our next step was to determine whether mGluR5 and CB_1_ employ similar mechanisms to trigger neuroprotection. Decreased release of glutamate by glutamatergic pre-synaptic sites was proposed to be one of the neuroprotective mechanisms elicited by CB_1_ activation [36, 52]. Thus, we investigated whether the neuroprotective drugs could diminish glutamate release. In the absence of glutamate insult, none of the tested drugs, CDPPB, URB597, JZL184, MPEP and AM251, modified glutamate release, as compared to basal levels (Fig. 2). Moreover, in the presence of glutamate, CDPPB (F_7,28_ = 30.84, *p* <0.0001; Fig. 2a), URB597 (F_7,28_ = 26.50, *p* <0.0001; Fig. 2b) and JZL184 (F_7,27_ = 39.25, *p* <0.0001; Fig. 2c) were also not efficient to modify extracellular glutamate levels. It has been shown that endocannabinoids can decrease glutamate release. However, as the cell death insult used in this study was glutamate itself, it was likely that decreasing glutamate release would not play a major role in the neuroprotective mechanism. We also measured intracellular Ca^2+^ levels in these neuronal cultures, as one possible cell survival mechanism would be to decrease the intracellular levels of this ion. Similarly, none of the tested drugs increased intracellular Ca^2+^ above basal levels in the absence of glutamate (Fig. 3). Incubation of neuronal cultures with glutamate induced high levels of intracellular Ca^2+^ (Fig. 3), which could contribute to neuronal excitotoxicity. However, CDPPB (F_7,54_ = 8.259, *p* <0.0001; Fig. 3a), URB597 (F_7,54_ = 8.300, *p* <0.0001; Fig. 3b) and JZL184 (F_7,54_ = 9.179, *p* <0.0001; Fig. 3c) did not modify glutamate-induced increased levels of intracellular Ca^2+^. Therefore, the cell survival mechanism elicited by CDPPB, URB597 and JZL184 in neurons treated with glutamate did not appear to involve either reduced glutamate release or decreased intracellular Ca^2+^ levels.

**Fig. 4:**
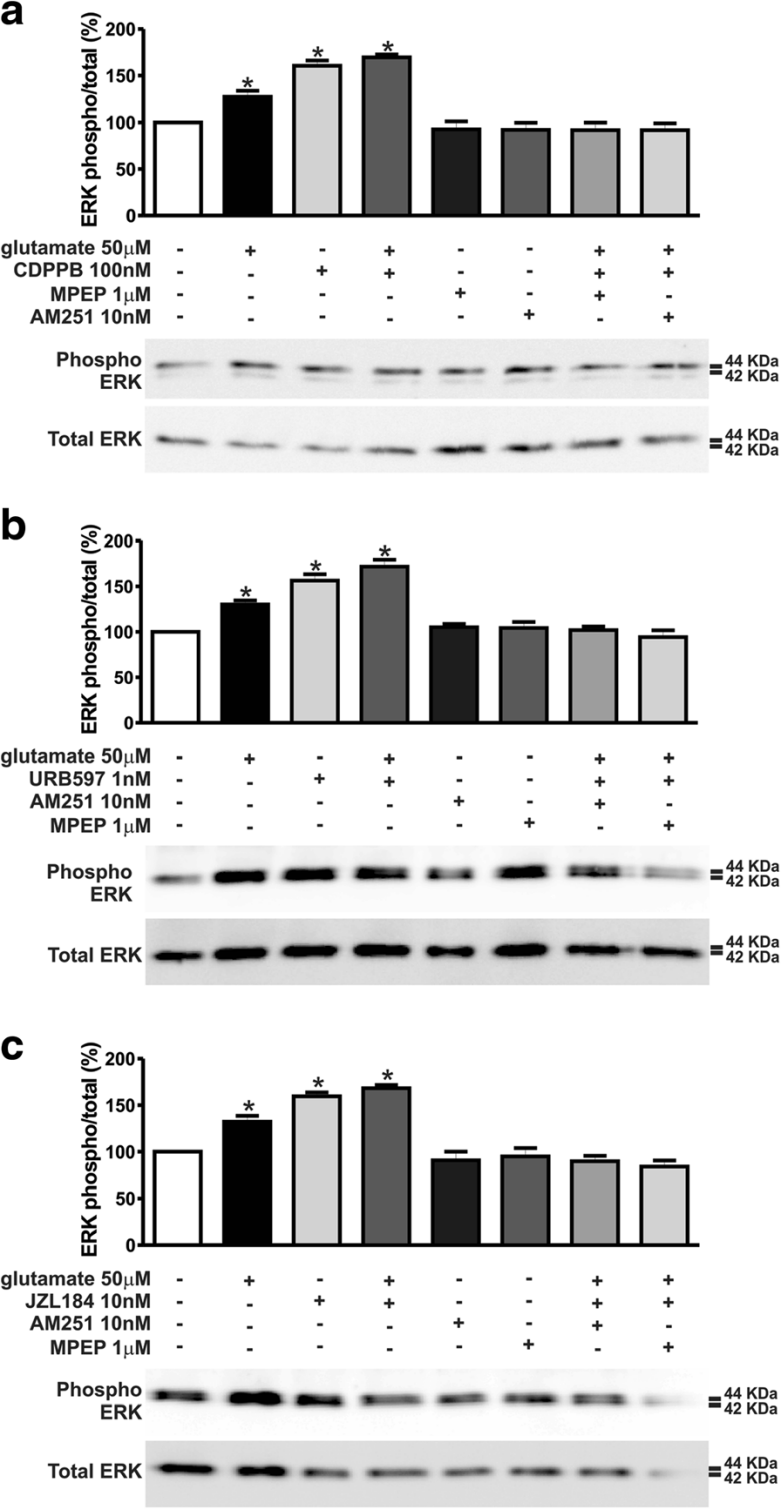
CDPPB-, URB597- and JZL184–mediated ERK1/2 phosphorylation is dependent on both mGluR5 and CB_1_. Shown are representative immunoblots for phospho- (upper panel) and total-ERK1/2 expression (lower panel) and graphs depicting the densitometric analysis of phospho-ERK1/2 normalized to total- ERK1/2 expression in primary cultured corticostriatal neurons that were either untreated (−) or treated (+) with 50 μM glutamate, 1 μM MPEP, 10 nM AM251, 100 nM CDPPB (**a**), 1 nM URB597 (**b**) and 10 nM JZL184 (**c**) for 7.5 min. 100 μg of cell lysate was used for each sample. Data represent the means ± SEM of four independent experiments. * indicates significant difference as compared to untreated neurons (*p* <0.05)

**Fig. 5:**
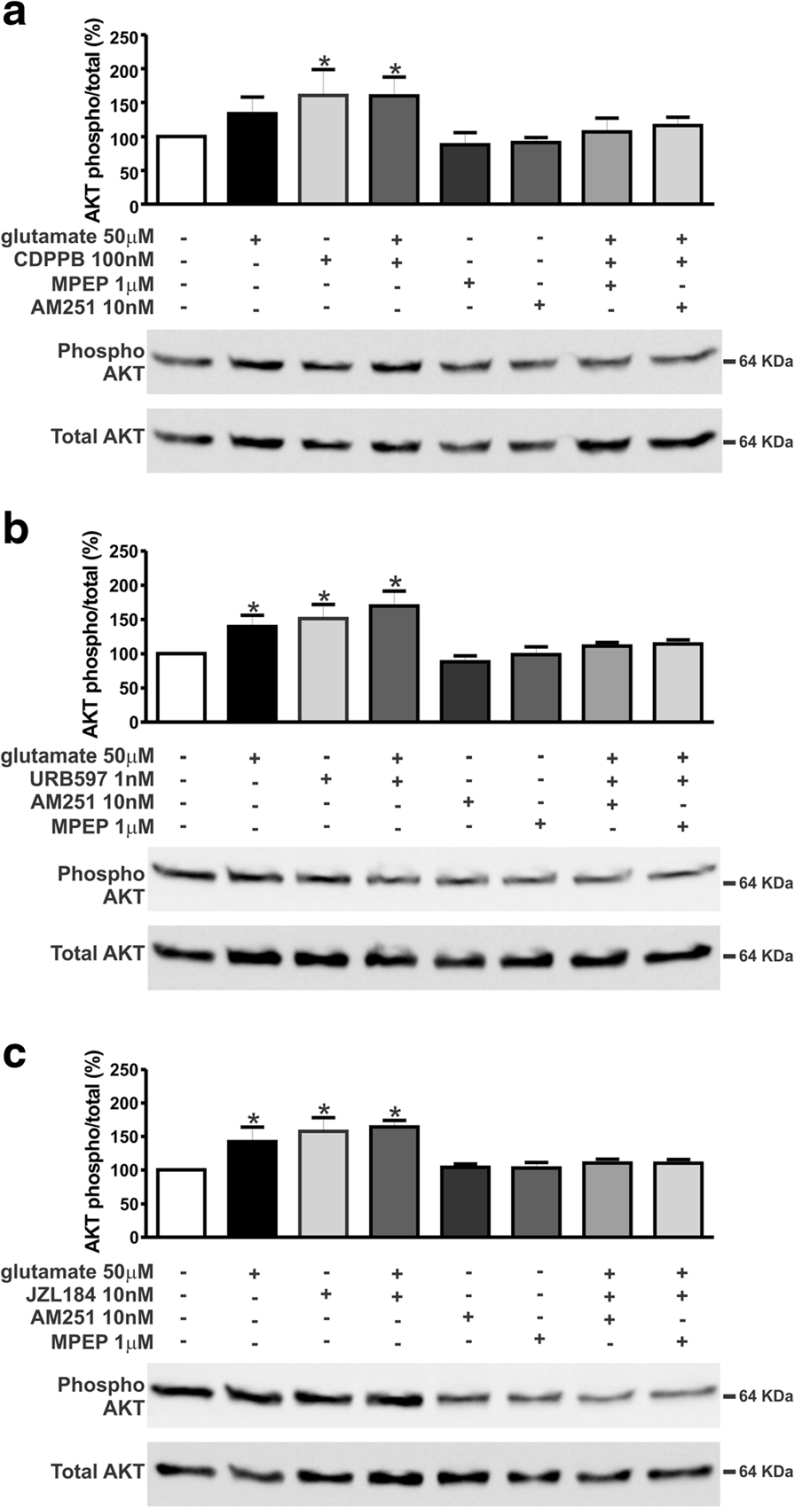
CDPPB-, URB597- and JZL184–mediated AKT phosphorylation requires both mGluR5 and CB_1_. Shown are representative immunoblots for phospho- (upper panel) and total-AKT expression (lower panel) and graphs depicting the densitometric analysis of phospho-AKT normalized to total-AKT expression in primary cultured corticostriatal neurons that were either untreated (−) or treated (+) with 50 μM glutamate, 1 μM MPEP, 10 nM AM251, 100 nM CDPPB (**a**), 1 nM URB597 (**b**) and 10 nM JZL184 (**c**) for 7.5 min. 100 μg of cell lysate was used for each sample. Data represent the means ± SEM of four independent experiments. * indicates significant difference as compared to untreated neurons (*p* <0.05)

As both mGluR5 and CB_1_ can activate similar cell survival pathways, we investigated whether activation of ERK1/2 and AKT could be involved in the mGluR5/CB_1_ neuroprotective mechanism. Stimulation of corticostriatal neuronal cultures with CDPPB was efficient to promote phosphorylation and thus activation of both ERK1/2 (F_7,24_ = 25.43, *p* < 0.0001; Fig. 4a) and AKT (F_7,24_ = 7.083, *p* = 0.0001; Fig. 5a) above basal levels, either in the absence or presence of glutamate. Both MPEP and AM251 were efficient to block ERK1/2 (Fig. 4a) and AKT (Fig. 5a) activation, suggesting that stimulation of mGluR5 by CDPPB requires CB_1_ activation. Moreover, URB597 and JZL184 were also efficient to activate ERK1/2 (F_7,24_ = 27.03, *p* <0.0001; Fig. 4b and F_7,24_ = 29.12, *p* <0.0001; c) and AKT (F_7,24_ = 18.37, *p* <0.0001; Fig. 5b and F_7,24_ = 19.68, *p* <0.0001; 5C) above basal levels, either in the absence or presence of glutamate. As in the case of CDPPB, both MPEP and AM251 eliminated ERK1/2 (Fig. 4b and c) and AKT (Fig. 5b and c) activation by URB597 and JZL184. Together, these data suggested that both mGluR5 and CB_1_ were necessary for the activation of important neuronal survival cell signaling pathways.

### CDPPB, URB597 and JZL184 treatment are unable to activate ERK1/2 and AKT and promote neuroprotection of either mGluR5^−/−^ or CB1 knockdown neurons

**Fig. 6:**
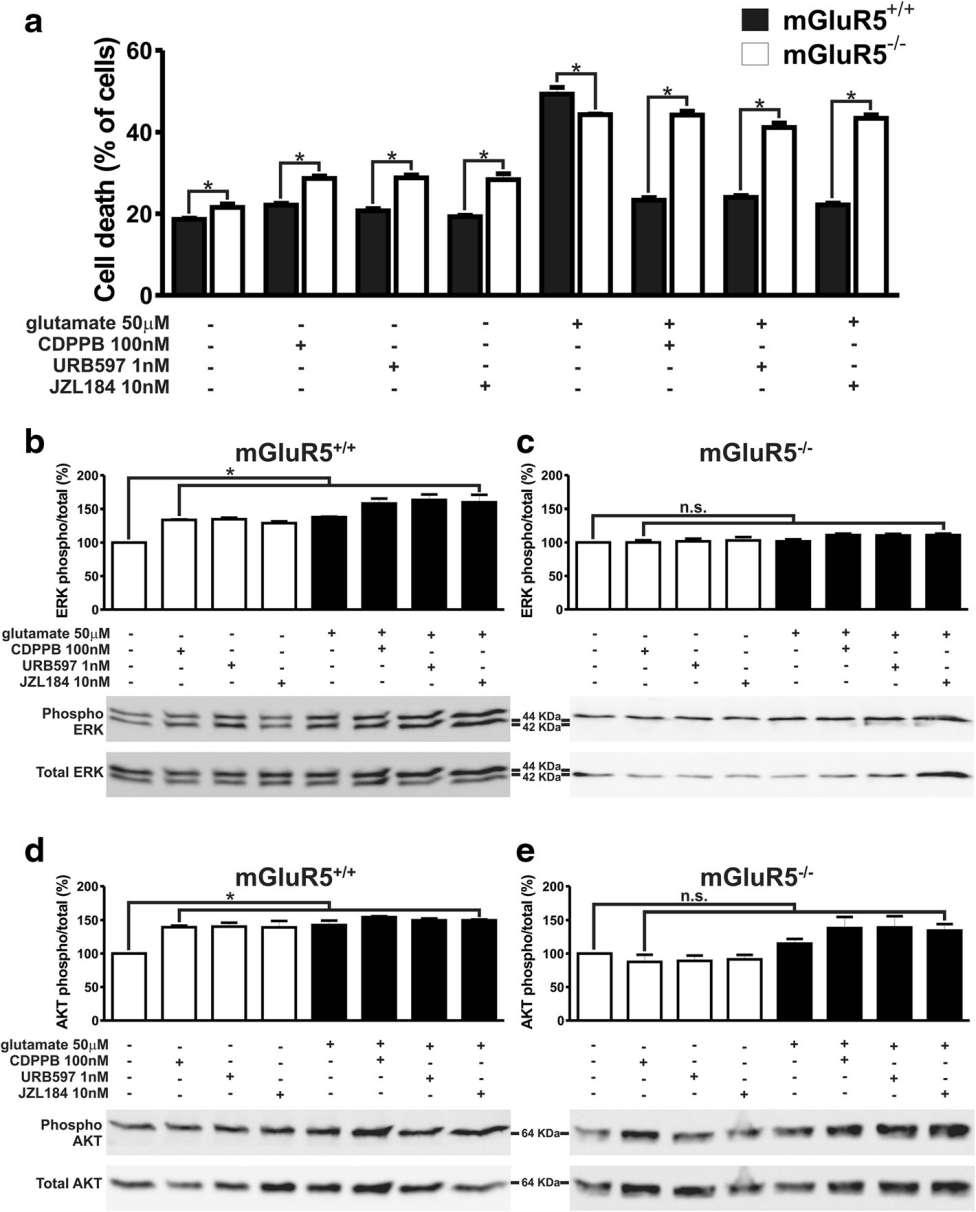
CDPPB, URB597 and JZL184 treatment are unable to activate ERK1/2 and AKT and promote neuroprotection of mGluR5^−/−^ neurons. **a** Graph shows cell death levels of corticostriatal neurons from either mGluR5^+/+^ or mGluR5^−/−^ embryos that were either untreated (−) or treated (+) with 50 μM glutamate, 100 nM CDPPB, 1 nM URB597 and 10 nM JZL184 for 4 h. Data represent the means ± SEM of four independent experiments. * indicates significant difference as compared to matched treated mGluR5^+/+^ neurons (*p* <0.05). Shown are representative immunoblots for phospho- (*upper* panel) and total-ERK1/2 expression (*lower* panel) and graphs depicting the densitometric analysis of phospho-ERK1/2 normalized to total- ERK1/2 expression in primary cultured corticostriatal neurons from either mGluR5^+/+^ (**b**) or mGluR5^−/−^ (**c**) embryos that were either untreated (−) or treated (+) with 50 μM glutamate, 100 nM CDPPB, 1 nM URB597 and 10 nM JZL184 for 7.5 min. Also shown are representative immunoblots for phospho- (*upper* panel) and total-AKT expression (*lower* panel) and graphs depicting the densitometric analysis of phospho-AKT normalized to total-AKT expression in primary cultured corticostriatal neurons from either mGluR5^+/+^ (**d**) or mGluR5^−/−^ (**e**) embryos that were either untreated (−) or treated (+) with 50 μM glutamate, 100 nM CDPPB, 1 nM URB597 and 10 nM JZL184 for 7.5 min. 100 μg of cell lysate was used for each sample. Data represent the means ± SEM of four independent experiments. n.s. indicates not significant and * indicates significant difference as compared to untreated neurons (*p* <0.05)

To determine whether the results obtained following pharmacological blockade of the receptors could be reproduced by genetic knockout, we employed primary cultured corticostriatal neurons obtained from mGluR5^−/−^ embryos to test the neuroprotective drugs. Interestingly, basal neuronal cell death was already higher in the case of mGluR5^−/−^ neurons, as compared to that of mGluR5^+/+^ neurons (F_7,72_ = 194.00, *p* <0.0001; Fig. 6a). Moreover, treatment with CDPPB, URB597 and JZL184, in the absence of glutamate, elicited higher levels of neuronal cell death in mGluR5^−/−^ neurons than in mGluR5^+/+^ neurons (Fig. 6a). However, glutamate insult promoted higher levels of neuronal cell death in mGluR5^+/+^ neurons than in mGluR5^−/−^ neurons (Fig. 6a). These data further highlighted the vital role of mGluR5 in cell death mechanisms. Importantly, although CDPPB, URB597 and JZL184 promoted neuroprotection against glutamate insult in mGluR5^+/+^ neurons, these drugs failed to promote survival of mGluR5^−/−^ neurons (Fig. 6a). In addition, CDPPB, URB597 and JZL184 were only able to activate ERK1/2 and AKT in mGluR5^+/+^ neurons (F_7,24_ = 32.95, *p* <0.0001; Fig. 6b and F_7,24_ = 14.58, *p* <0.0001; d), but not in mGluR5^−/−^ neurons (F_7,32_ = 2.674, *p* = 0,0262; Fig. 6c and F_7,24_ = 4.475, *p* = 0.0026; e).

**Fig. 7:**
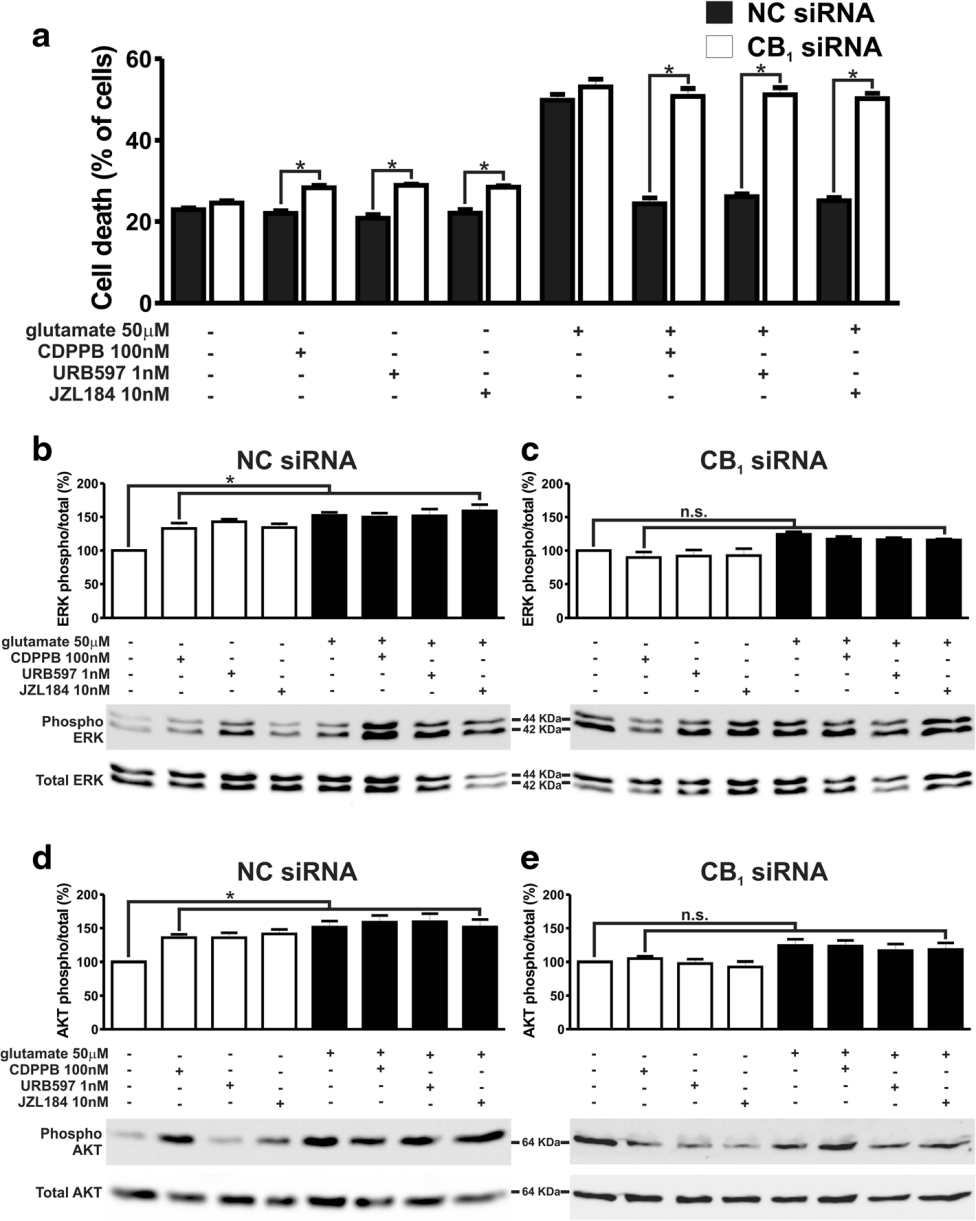
CDPPB, URB597 and JZL184 treatments fail to activate ERK1/2 and AKT and promote neuroprotection of CB_1_ knockdown neurons. **a** Graph shows cell death levels of corticostriatal neurons that were electroporated with either NC- or CB_1_-siRNA and that were either untreated (−) or treated (+) with 50 μM glutamate, 100 nM CDPPB, 1 nM URB597 and 10 nM JZL184 for 4 h. Data represent the means ± SEM of four independent experiments. * indicates significant difference as compared to matched treated NC-siRNA electroporated neurons (*p* <0.05). Shown are representative immunoblots for phospho- (*upper* panel) and total-ERK1/2 expression (*lower* panel) and graphs depicting the densitometric analysis of phospho-ERK1/2 normalized to total-ERK1/2 expression in primary cultured corticostriatal neurons that were electroporated with either NC- (**b**) or CB_1_-siRNA (**c**) and that were either untreated (−) or treated (+) with 50 μM glutamate, 100 nM CDPPB, 1 nM URB597 and 10 nM JZL184 for 7.5 min. Also shown are representative immunoblots for phospho- (upper panel) and total-AKT expression (lower panel) and graphs depicting the densitometric analysis of phospho-AKT normalized to total-AKT expression in primary cultured corticostriatal neurons that were electroporated with either NC- (**d**) or CB_1_-siRNA (**e**) and that were either untreated (−) or treated (+) with 50 μM glutamate, 100 nM CDPPB, 1 nM URB597 and 10 nM JZL184 for 7.5 min. 100 μg of cell lysate was used for each sample. Data represent the means ± SEM of four independent experiments. n.s. indicates not significant and * indicates significant difference as compared to untreated neurons (*p* <0.05)

To determine whether a decrease in CB_1_ protein expression could compromise neuroprotection via mGluR5 and CB_1_, we electroporated CB_1_ small interfering RNA (siRNA) into corticostriatal neurons to knockdown CB_1_ expression. A negative control (NC) siRNA, which did not target murine mRNAs, was also used. CB_1_-siRNA efficiently knockdown CB_1_ protein expression by 55 %, as compared to NC-siRNA (Additional file 2: Figure S2). Basal neuronal cell death levels were not different when comparing neurons electroporated with either CB_1_-siRNA or NC-siRNA (F_7,48_ = 157.4, *p* <0.0001; Fig. 7a). However, stimulation of neurons with CDPPB, URB597 and JZL184, in the absence of glutamate, triggered higher cell death levels of CB_1_ knockdown neurons than of control neurons (Fig. 7a). These data indicated that compromised expression of CB_1_ could render neurons more sensitive to the toxic effects of drugs, further supporting the premise that CB_1_ plays a key role in cell survival mechanisms. Glutamate insult increased cell death levels to the same extent when comparing NC-siRNA or CB_1_-siRNA electroporated neurons (Fig. 7a). As in the case of mGluR5^−/−^, CDPPB, URB597 and JZL184 were not able to protect CB_1_ knockdown neurons against glutamate insult, although these drugs were efficient to protect control neurons exposed to glutamate (Fig. 7a). Moreover, CDPPB, URB597 and JZL184 were only capable of activating ERK1/2 and AKT in control neurons (F_7,24_ = 7.929, *p* <0.0001; Fig. 7b and F_7,24_ = 5.252, *p* = 0,0010; d), but not in CB_1_ knockdown neurons (F_7,24_ = 5.231, *p* = 0.0010; Fig. 7c and F_7,24_ = 2.649, *p* = 0.0352; e). These data further support the hypothesis that mGluR5 and CB_1_ act in a cooperative manner to activate cell signaling pathways that lead to neuroprotection.

### Neuroprotection induced by CDPPB, URB597 and JZL184 is dependent on both ERK1/2 and AKT

**Fig. 8:**
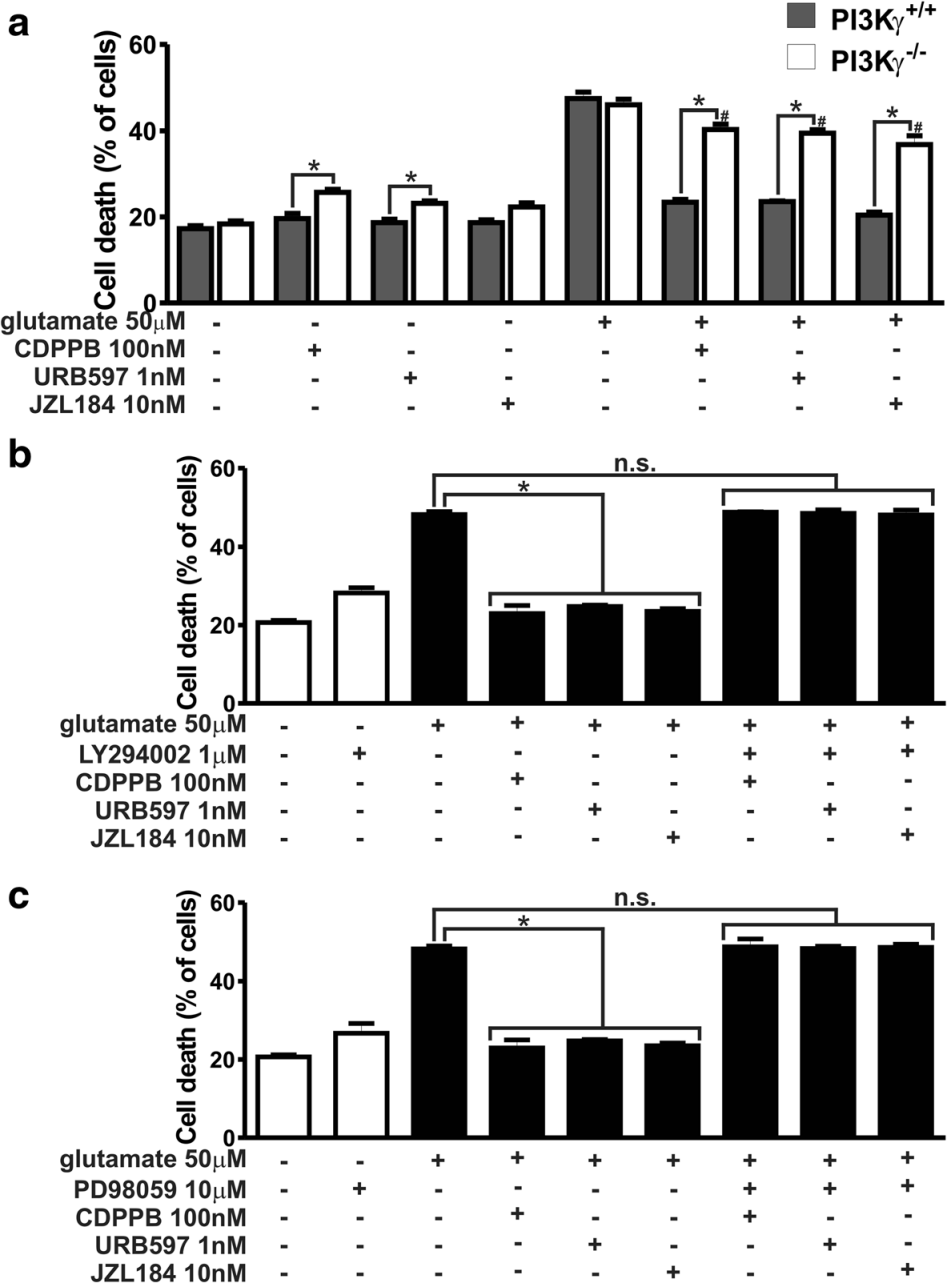
Neuroprotection induced by CDPPB, URB597 and JZL184 is dependent on both ERK1/2 and AKT. **a** Graph shows cell death levels of corticostriatal neurons from either PI3Kγ^+/+^ or PI3Kγ^−/−^ embryos that were either untreated (−) or treated (+) with 50 μM glutamate, 100 nM CDPPB, 1 nM URB597 and 10 nM JZL184 for 4 h. Data represent the means ± SEM of four independent experiments. * indicates significant difference as compared to matched treated PI3Kγ^+/+^ neurons and # indicates significant difference as compared to glutamate-treated PI3Kγ^−/−^ neurons (*p* <0.05). Graphs show cell death levels of primary cultured corticostriatal neurons that were either untreated (−) or treated (+) with 50 μM glutamate, 100 nM CDPPB, 1 nM URB597, 10 nM JZL184, 1 μM LY294002 (**b**) and 10 μM PD98059 (**c**) for 4 h. Data represent the means ± SEM of four independent experiments. n.s. indicates not significant and * indicates significant difference as compared to glutamate treated neurons (*p* <0.05)

To determine whether AKT was indeed necessary for CB_1_ and mGluR5 neuroprotection, we performed cell death experiments in corticostriatal neurons obtained from mice knockout for the phosphatidylinositol-3 kinase γ (PI3Kγ^−/−^), as PI3K is a key component that is upstream from AKT activation by mGluRs and CB_1_ [15, 50]. In the absence of glutamate, PI3Kγ^−/−^ neurons incubated with CDPPB and URB597 exhibited higher levels of neuronal cell death than those of PI3Kγ^+/+^ neurons (F_7,64_ = 175.3, *p* <0.0001; Fig. 8a), possibly because PI3Kγ knockout decreased activation of cell survival pathways and made neurons more prone to toxicity. Although CDPPB, URB597 and JZL184 promoted neuroprotection of PI3Kγ^+/+^ neurons under the insult of glutamate, neuroprotection triggered by these drugs was partially abrogated in PI3Kγ^−/−^ neurons incubated with glutamate (Fig. 8a). Moreover, CDPPB, URB597 and JZL184 failed to activate AKT in PI3Kγ^−/−^ neurons (F_7,16_ = 7.03, *p* = 0.0006; Additional file 3: Figure S3). However, in the presence of glutamate, CDPPB, URB597 and JZL184 were still able to increase AKT phosphorylation above basal levels in PI3Kγ^−/−^ neurons (Additional file 3: Figure S3). It has been shown that GPCRs can activate AKT through both PI3Kγ and PI3Kβ and that these kinases may thus have a redundant function [17]. Therefore, it was possible that other PI3K isoforms, including PI3Kβ, could also contribute to AKT activation when neurons were stimulated with the tested drugs in the presence of glutamate, which could explain why neuroprotection was not completely eliminated in PI3Kγ^−/−^ neurons. To further investigate the role of AKT activation in CDPPB-, URB597- and JZL184-mediated neuroprotection, we pre-incubated neurons with 1 μM of LY294002, which inhibited all PI3K isoforms that were relevant to neuroprotective mechanisms [64]. LY294002 completely abrogated neuronal protection triggered by CDPPB, URB597 and JZL184 (F_8,27_ = 91.34, *p* <0.0001; Fig. 8b), strongly indicating that the PI3K/AKT pathway was necessary for CB_1_- and mGluR5-mediated neuroprotection. To determine whether ERK1/2 activation was also important for mGluR5/CB_1_-mediated neuroprotection, we inhibited the mitogen-activated protein kinase kinase (MEK), the main kinase responsible for ERK1/2 activation. Inhibition of MEK using 10 μM PD98059 eliminated the neuroprotection prompted by CDPPB, URB597 and JZL184 (F_8,27_ = 155.7, *p* <0.0001; Fig. 8c). Taken together, these data strongly indicated that CB_1_ and mGluR5 mediated neuroprotection depends on both PI3K/AKT and MEK/ERK1/2 pathways.

### CDPPB protects the postsynaptic site and JZL184 mainly protects presynaptic terminals

**Fig. 9:**
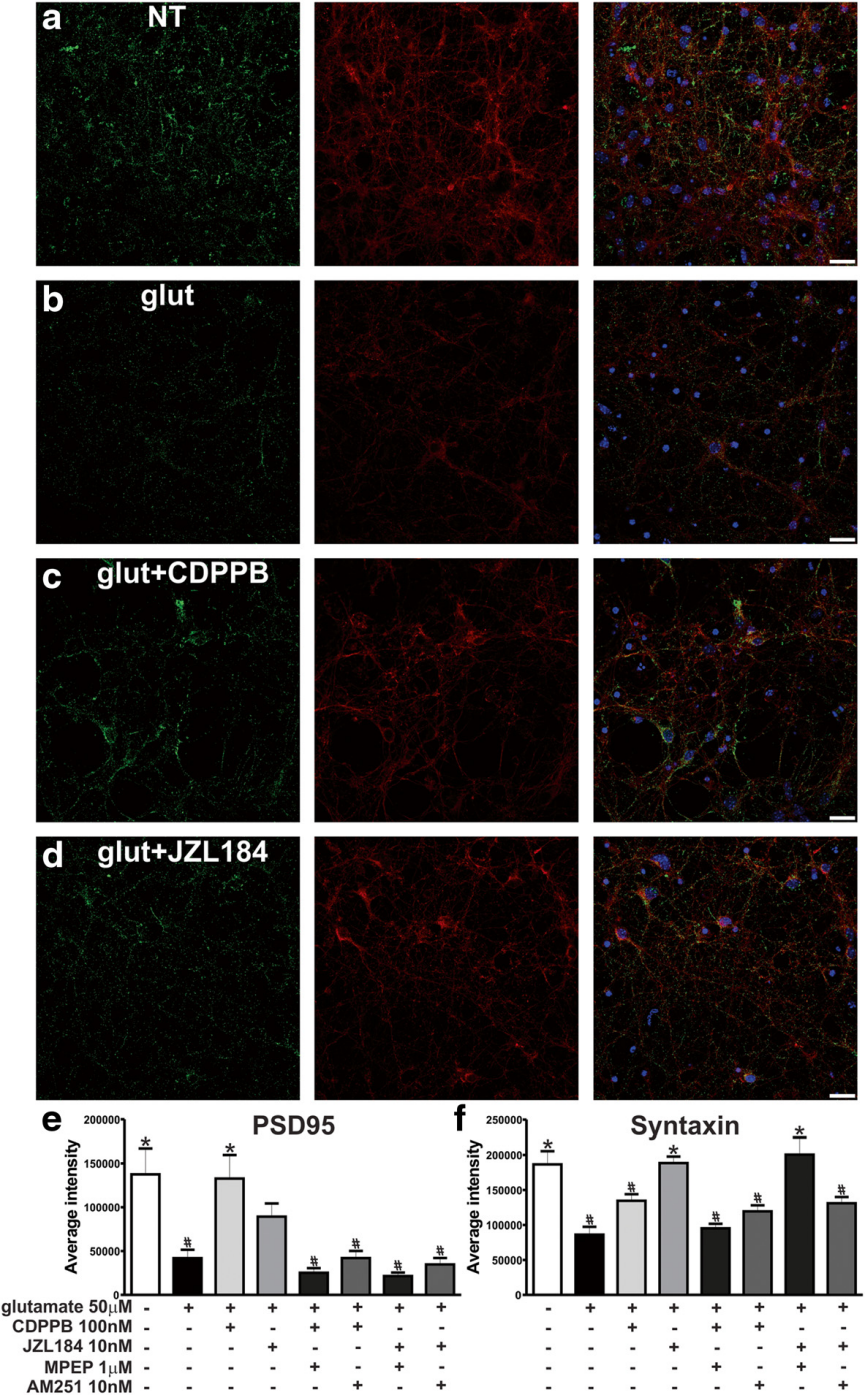
CDPPB protects postsynaptic terminals and JZL184 mainly protects the presynaptic site. Shown are laser-scanning confocal micrographs from neurons immunolabeled for PSD95 (*green*), anti-syntaxin 1A (*red*) and DAPI (*blue*), which were either untreated (NC) (**a**) or treated with 50 μM glutamate (glut) in the absence (**b**) or in the presence of 100 nM CDPPB (**c**) and 10 nM JZL184 (**d**) for 4 h. Scale bar = 20 μM. Graphs show average intensity of pixel grey levels of PSD95 (**e**) and syntaxin 1A (**f**) staining, obtained from at least five images taken from four independent experiments. Neurons were either untreated (−) or treated (+) with 50 μM glutamate, 100 nM CDPPB, 10 nM JZL184, 1 μM MPEP or 10 nM AM251, for 4 h. Data represent the means ± SEM. * indicates significant difference as compared to glutamate treated neurons and # indicates significant difference as compared to untreated neurons (*p* <0.05)

The loss of synaptic terminals can be regarded as the most important feature of neurodegeneration as synaptic loss better correlates with disease progression than neuronal cell loss itself [19, 59]. mGluR5 is mostly expressed at the post-synaptic site and CB_1_ is mainly pre-synaptic [29, 53]. Thus, we decided to investigate whether mGluR5 and CB_1_ activation could prevent the loss of pre- and/or post-synaptic terminals following glutamate insult. To test this, we treated neurons with the previously tested drugs and performed immunofluorescence experiments to label the postsynaptic density protein, PSD95, and the presynaptic marker, syntaxin 1A, and then quantified the levels of post- and pre-synaptic loss. 50 μM glutamate promoted a significant decrease in PSD95 and syntaxin 1A labeling (Fig. 9b, F_7,203_ = 7.765, *p* <0.0001; e and F_7,198_ = 11.01, *p* <0.0001; f), as compared to untreated neurons (Fig. 9a, e and f), indicating that this neurotransmitter could trigger the loss of both post- and pre-synaptic terminals. When neurons were incubated with glutamate in the presence of CDPPB, the loss of PSD95 labeling was rescued (Fig. 9c and e). Although CDPPB augmented syntaxin levels, this increase was not significantly different than that of neurons incubated with glutamate (Fig. 9c and f). Both MPEP (Additional file 4: Figure S4A and Fig. 9e) and AM251 (Additional file 4: Figure S4B and Fig. 9e) eliminated CDPPB-mediated rescuing of postsynaptic sites. These data indicated that CDPPB was more efficient to protect postsynaptic terminals and that this neuroprotection was dependent on both mGluR5 and CB_1_. JZL184 was efficient to prevent glutamate-induced decrease in syntaxin labeling (Fig. 9d and f). Moreover, JZL184 was partially efficient to avoid the loss of postsynaptic terminals, as PSD95 labeling of neurons incubated with JZL184 in the presence of glutamate was not different than that of untreated neurons (Fig. 9d and e). However, PSD95 labeling was not different when comparing neurons incubated with glutamate in the presence or absence of JZL184 (Fig. 9d and e). AM251 eliminated the protection of both pre- and post-synaptic terminals mediated by JZL184 (Additional file 4: Figure S4D, Fig. 9e and f). Nevertheless, MPEP was only able to block JZL184-mediated rescuing of postsynaptic terminals, having no effect on syntaxin levels (Additional file 4: Figure S4C, Fig. 9e and f). These data indicated that 2-AG was more important to promote neuroprotection of presynaptic sites and that CB_1_ and mGluR5 had distinct roles depending on which synaptic site was being investigated.

## Discussion

Neuronal cell loss is the main feature of neurodegenerative diseases and the development of new drugs capable of preventing neuronal cell death might be an important therapeutic strategy to modify the course of these diseases. However, probably due to the complexity of the cell signaling pathways that underlie neuronal cell loss and neuroprotection of brain cells, such a disease-modifying drug is yet to be discovered [14, 55]. CB_1_ and mGluR5 are promising pharmacological targets for the development of neuroprotective drugs. For instance, CB1 activation offer protection in various neurodegenerative diseases, including Alzheimer’s disease [37, 38, 56], Parkinson’s disease [30, 46], and HD [39, 41]. In the case of mGluR5, recent publications consistently indicate that mGluR5 PAMs can be neuroprotective in acute brain injury [7, 32, 68]. Moreover, our group has demonstrated that the mGluR5 PAM, CDPPB, can promote neuroprotection in vitro and that the chronic treatment of a mouse model of HD with CDPPB can prevent neuronal cell loss, decrease huntingtin aggregate formation and rescue memory deficit [10, 11].

CB_1_ activation can decrease pre-synaptic release of neurotransmitters, including glutamate [44, 66]. Decreased glutamate release has been proposed as the key factor contributing to CB_1_-mediated neuroprotection [28, 36]. However, CB_1_-mediated neuroprotection did not involve decreased glutamate release in the present protocol. On the other hand, our data clearly shows that neuroprotection triggered by CB_1_ and mGluR5 depends on both ERK1/2 and AKT activation. CB_1_ and mGluR5 can activate these kinases employing different mechanisms. Stimulation of mGluRs promotes the formation of the complex mGluR-Homer-PI3K enhancer (PIKE), which allows PI3K activation by PIKE, leading to PIP3 formation, which recruits AKT and phosphoinositide-dependent kinase (PDK1) to the plasma membrane to promote AKT phosphorylation [21, 50]. Thus, activation of AKT by mGluR5 appears to be independent of Gα_q_ proteins. Homer proteins are also important for mGluR5-mediated activation of ERK1/2 in the striatum and spinal cord [35, 49, 58]. Moreover, it has been shown that the proline-rich tyrosine kinase 2 (Pyk2) can be co-immunoprecipitated with both mGluR1 and mGluR5 from rat brain lysates and that Pyk2 can couple Group I mGluRs to the activation of ERK1/2 [43]. Importantly, we and others have demonstrated that mGluR5 PAMs can act as bias agonists and activate ERK1/2 and AKT, in the absence of agonist and without triggering Ca^2+^ release [5, 7, 11, 67], which was further demonstrated on this study. In the case of the cannabinoid system, it has been shown that stimulation of CB_1_ can lead to activation of PI3K/AKT pathways in a mechanism that relies on G_αio_ proteins [15]. Moreover, ERK1/2 can be activated by CB_1_ heterologously expressed in non-neuronal cell lines [4, 65]. In addition to that, it has also been demonstrated that stimulation of CB_1_ can lead to ERK1/2 activation in hippocampal neurons and that PKA inhibition via uncoupling of Gα_io_ proteins by CB_1_ is important for ERK1/2 activation [8]. Importantly, activation of these cell signaling pathways by mGluR5 and CB_1_ can lead to increased levels of trophic factors, including brain derived neurotrophic factor (BDNF), which has an important role in neuronal survival [2, 8, 10].

CB_1_ is mostly expressed at the presynaptic site and mGluR5 at the postsynaptic region. Nevertheless these two receptors have a functional interaction, as CB_1_ can regulate presynaptic glutamate release [26, 44, 66] and mGluR5 activation can increase endocannabinoid synthesis at the postsynaptic site [6, 23, 24]. However, so far it is still unclear whether mGluR5 activation could preferentially protect the post-synaptic site or whether CB_1_ is more neuroprotective at the pre-synaptic area. Our data demonstrates that CDPPB offers more protection to the postsynaptic site and JZL184 protects more the presynaptic terminals. CDPPB-mediated protection of postsynaptic terminals is lost when either mGluR5 or CB_1_ is blocked. Thus, it is possible that one of the mechanisms employed by mGluR5 to promote neuroprotection is to activate CB_1_, which may be accomplished by increasing 2-AG synthesis following mGluR5 activation. JZL184 is very efficient to promote neuroprotection of presynaptic terminals and also offers partial rescuing of postsynaptic sites. In agreement with these data, the rescue of CB_1_ expression in striatal neurons of a mouse model of HD prevents the reduction of excitatory presynaptic markers [41]. Importantly, we show in this study that MPEP is only able to block JZL184 protection of the postsynaptic terminals, but not of the presynaptic site. It is possible that the main role of mGluR5 in JZL184-mediated neuroprotection is to increase endocannabinoid synthesis, as inhibiting 2-AG degradation is not sufficient to elevate the levels of endocannabinoids to promote neuroprotection if mGluR5-mediated synthesis of 2-AG is blocked by MPEP. It has been demonstrated that disruption of the interaction between mGluR5 and DGL-α can compromise 2-AG production and long term depression (LTD) at excitatory synapses, further indicating that mGluR5 has a crucial role in 2-AG synthesis [25]. Corroborating this hypothesis, we show in this study that CB_1_ direct agonists could mediate neuroprotection to some extent independently of mGluR5. However, as MPEP is still able to partially block even CB_1_ direct agonist-mediated neuroprotection, it is possible that mGluR5 may have roles other than facilitate endocannabinoid synthesis to promote neuronal survival. Future experiments will be important to clarify this issue. Interestingly, mGluR5 appears to be important for JZL184-mediated neuroprotection only at the postsynaptic site, which is the main place where mGluR5-dependent 2-AG synthesis takes place. High levels of CB_1_ are expressed in the presynaptic site and it is possible that low levels of endocannabinoids could be sufficient to promote neuroprotection in this case. Moreover, although JZL184 prevents the loss of presynaptic terminals independently of mGluR5, this is not enough to avoid the neuronal cell loss, as JZL184 fails to rescue neuronal cell death upon glutamate insult in the presence of MPEP. Both CB_1_ and mGluR5 activate ERK1/2 and AKT and our data clearly show that this is the primary mechanism to promote neuroprotection. It has been previously demonstrated that ERK and AKT are present and can be phosphorylated/activated both in the pre- and post-synaptic terminals [3, 31, 54, 61]. Based on these observations, we propose that the protection of both pre- and post-synaptic sites, perhaps via activation of ERK1/2 and AKT in these two compartments, is necessary to preserve the synaptic structure and foster neuronal survival.

**Fig. 10:**
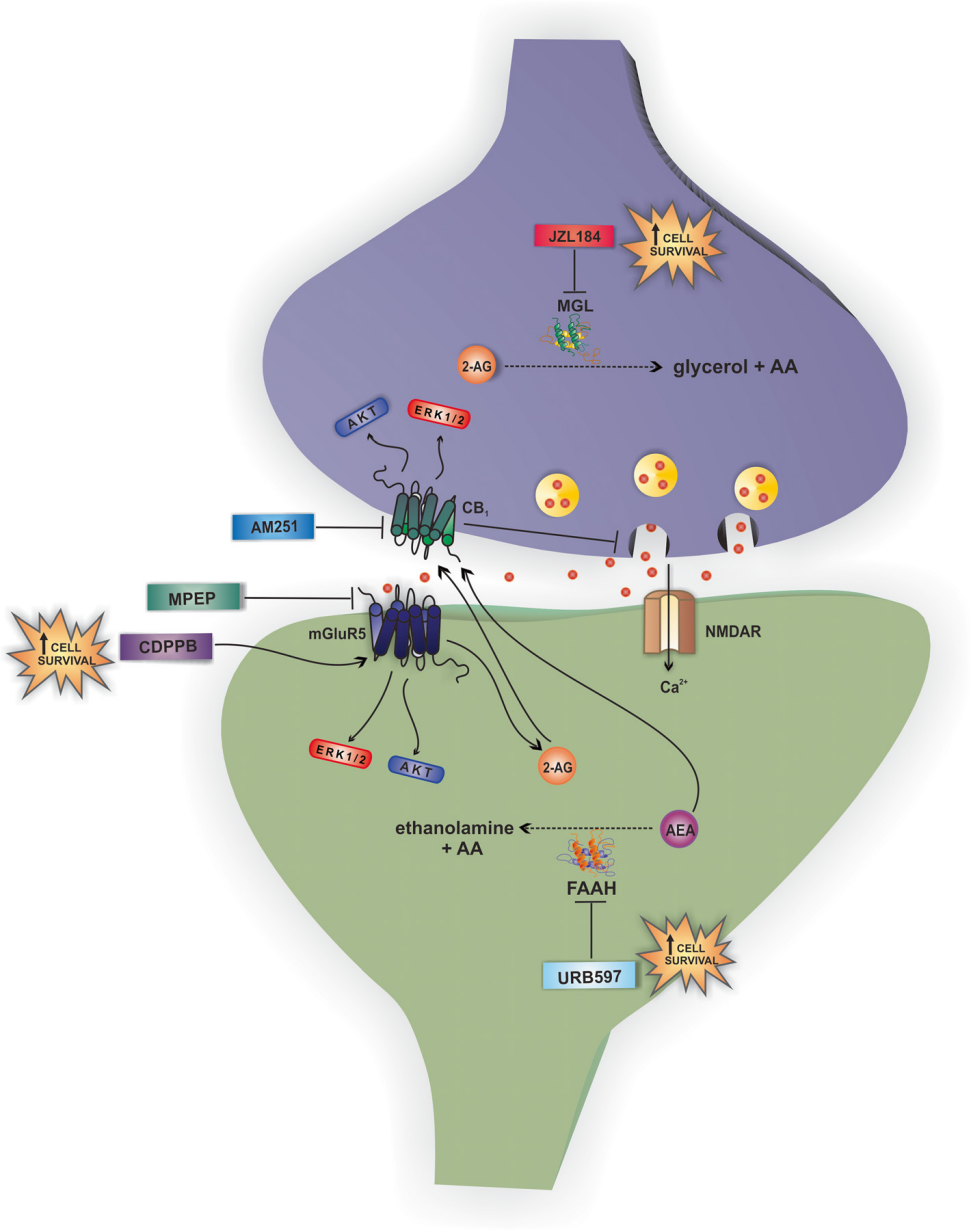
mGluR5 and CB_1_ cooperatively activate cell signaling pathways to promote neuroprotection. Glutamate release causes activation of N-methyl-D-aspartate receptor (NMDAR), which increases intracellular Ca^2+^, and metabotropic glutamate receptor 5 (mGluR5), which activates various cell signaling pathways. mGluR5 is present at the postsynaptic site and can be stimulated by CDPPB, leading to the activation of different neuroprotective effectors, such as the extracellular-signal-regulated kinase 1/2 (ERK1/2) and AKT. Moreover, mGluR5 is part of a signalosome that contains the two key enzymes for 2-arachidonoylglycerol (2-AG) synthesis and, thus, mGluR5 activation can increase 2-AG levels. However, both MPEP, an mGluR5 blocker, and AM251, a CB_1_ antagonist, can block mGluR5-mediated activation of ERK1/2 and AKT and, thus, neuroprotection. The cannabinoid receptor 1 (CB_1_), which is present at the presynaptic site, is stimulated by 2-AG and anandamide (AEA), leading to activation of extracellular-signal-regulated kinase (ERK1/2) and AKT. CB_1_ activation can also inhibit presynaptic glutamate release and, therefore, blunt mGluR5 activation by glutamate. JZL184 and URB597, which are monoacylglycerol lipase (MGL) and fatty acid amide hydrolase (FAAH) inhibitors, respectively, increase the levels of anandamide and 2-AG, leading to activation of ERK1/2 and AKT and promoting neuroprotection. However, this effect can be blocked by both MPEP and AM251. Therefore, mGluR5 and CB_1_ are part of the same cell signaling pathway, working cooperatively to trigger activation of ERK1/2 and AKT and promote neuroprotection. *AA* arachidonic acid

As summarized in “Fig. 10”, we show here that activation of mGluR5 by CDPPB or CB_1_ by blocking endocannabinoid degradation promotes neuroprotection against glutamate-induced excitotoxicity. Interestingly, if mGluR5 or CB_1_ are blocked pharmacologically or by genetic manipulation, the neuroprotection mediated by both receptors is lost, indicating that these two receptors are part of the same neuroprotective cell signaling pathway. In addition, our data demonstrate that the neuroprotection mediated by mGluR5/CB_1_ does not rely on decreased pre-synaptic release of glutamate or decreased intracellular Ca^2+^ levels, but instead is dependent on the activation of MEK/ERK1/2 and PI3K/AKT pathways.

## Additional files

Additional file 1: Figure S1. Cannabinoid receptors direct agonists can promote neuroprotection in an mGluR5-independent manner. Graph shows cell death levels of primary cultured corticostriatal neurons that were either untreated (−) or treated (+) with 50 μM glutamate, 10 nM anandamide, 10 nM 2-AG, 1 nM ACEA, 1 μM MPEP and 10 nM AM251 for 4 h. Data represent the means ± SEM of four independent experiments. * indicates significant difference as compared to glutamate treated neurons and # indicates significant difference as compared to untreated neurons (*p* <0.05). (TIF 270 kb) Additional file 2: Figure S2. CB_1_ siRNA electroporation decreases CB_1_ protein expression. Shown are representative immunoblots for CB_1_ (upper panel) and actin expression (lower panel) in primary cultured corticostriatal neurons that were electroporated with either NC- or CB_1_-siRNA. Immunoblots are representative of four independent experiments. (TIF 292 kb) Additional file 3: Figure S3. AKT activation is decreased, but not abolished, in PI3Kγ^−/−^ neurons. Shown are representative immunoblots for phospho- (upper panel) and total-AKT expression (lower panel) and graphs depicting the densitometric analysis of phospho-AKT normalized to total-AKT expression in primary cultured corticostriatal neurons from PI3Kγ^−/−^ embryos that were either untreated (−) or treated (+) with 50 μM glutamate, 100 nM CDPPB, 1 nM URB597 and 10 nM JZL184 for 7.5 min. 100 μg of cell lysate was used for each sample. Data represent the means ± SEM of four independent experiments. * indicates significant difference as compared to untreated neurons (*p* <0.05). (TIF 568 kb) Additional file 4: Figure S4. MPEP and AM251 action on CDPPB- and JZL184-mediated protection of synaptic terminals. Shown are laser-scanning confocal micrographs depicting Alexa Fluor 488-conjugated anti-PSD95 antibody (green), Alexa Fluor 546-conjugated anti-syntaxin 1A antibody (red) and DAPI (blue) in neurons that were treated with 50 μM glutamate (glut) in the presence of 100 nM CDPPB + 1 μM MPEP (A), 100 nM CDPPB + 10 nM AM251 (B), 10 nM JZL184 + 1 μM MPEP (C) and 10 nM JZL184 + 10 nM AM251 (D) for 4 h. Scale bar = 20 μM. (TIF 8576 kb)

## Abbreviations

2-AG: 2-arachidonoylglycerol
ACEA: Arachidonyl-2‘-Chloroethylamide
AM: Acetoxymethyl Ester
AM251: 1-(2,4-Dichlorophenyl)-5-(4-iodophenyl)-4-methyl-N-1-piperidinyl-1H-pyrazole-3-carboxamide
anandamide: N-arachidonoylethanolamine
APP: Amyloid Precursor Protein
Aβ: Amyloid-β Peptide
BDNF: Brain Derived Neurotrophic Factor
CB_1_: Cannabinoid Receptor 1
CDPPB: 3-cyano-*N*-(1,3-diphenyl-1*H*-pyrazol-5-yl)benzamide
DAG: Diacylghycerol
DGL-α: Diacylglycerol Lipase-α
DIV: Days In Vitro
ERK: Extracellular-Signal-Regulated Kinase
FAAH: Fatty Acid Amide Hydrolase
GABA: Gamma-Aminobutyric Acid
GPCR: G-protein Coupled Receptor
HBSS: Hank’s Balanced Salt Solution
HD: Huntington’s Disease
IP_3_: Inositol-1,4,5-triphosphate
JZL184: 4-[Bis(1,3-benzodioxol-5-yl)-hydroxymethyl]piperidine-1-carboxylate 4-nitrophenyl ester
LTD: Long Term Depression
LY294002: 2-(4-morpholinyl)-8-phenyl-1(4H)-benzopyran-4-one hydrochloride
MEK: Mitogen-Activated Protein Kinase Kinase
MGL: Monoacylglycerol Lipase
mGluR5: Metabotropic Glutamate Receptor 5
MPEP: 2-methyl-6-(phenylethynyl)-pyridine
NC: Negative Control
PAM: Positive Allosteric Modulator
PD98059: 2-(2-amino-3-methoxyphenyl)-4h-1-benzopyran-4-one
PDK1: Phosphoinositide-Dependent Kinase
PI3K: Phosphatidylinositol-3 kinase
PIKE: PI3K Enhancer
PLC: Phospholipase C
PSD95: Postsynaptic Density Protein
Pyk2: Proline-Rich Tyrosine Kinase 2
siRNA: Small Interfering RNA
URB597: Cyclohexyl Carbamic Acid 3‘-carbamoyl-biphenyl-3-yl ester
Δ^9^-THC: Δ^9^-tetrahydrocannabinol
