# Supplementary figures and images for: Orchestrated activation of mGluR5 and CB1 promotes neuroprotection
Source: Mol Brain. 2016 Aug 20;9:80. doi: 10.1186/s13041-016-0259-6 (PMC4992217; doi:10.1186/s13041-016-0259-6)

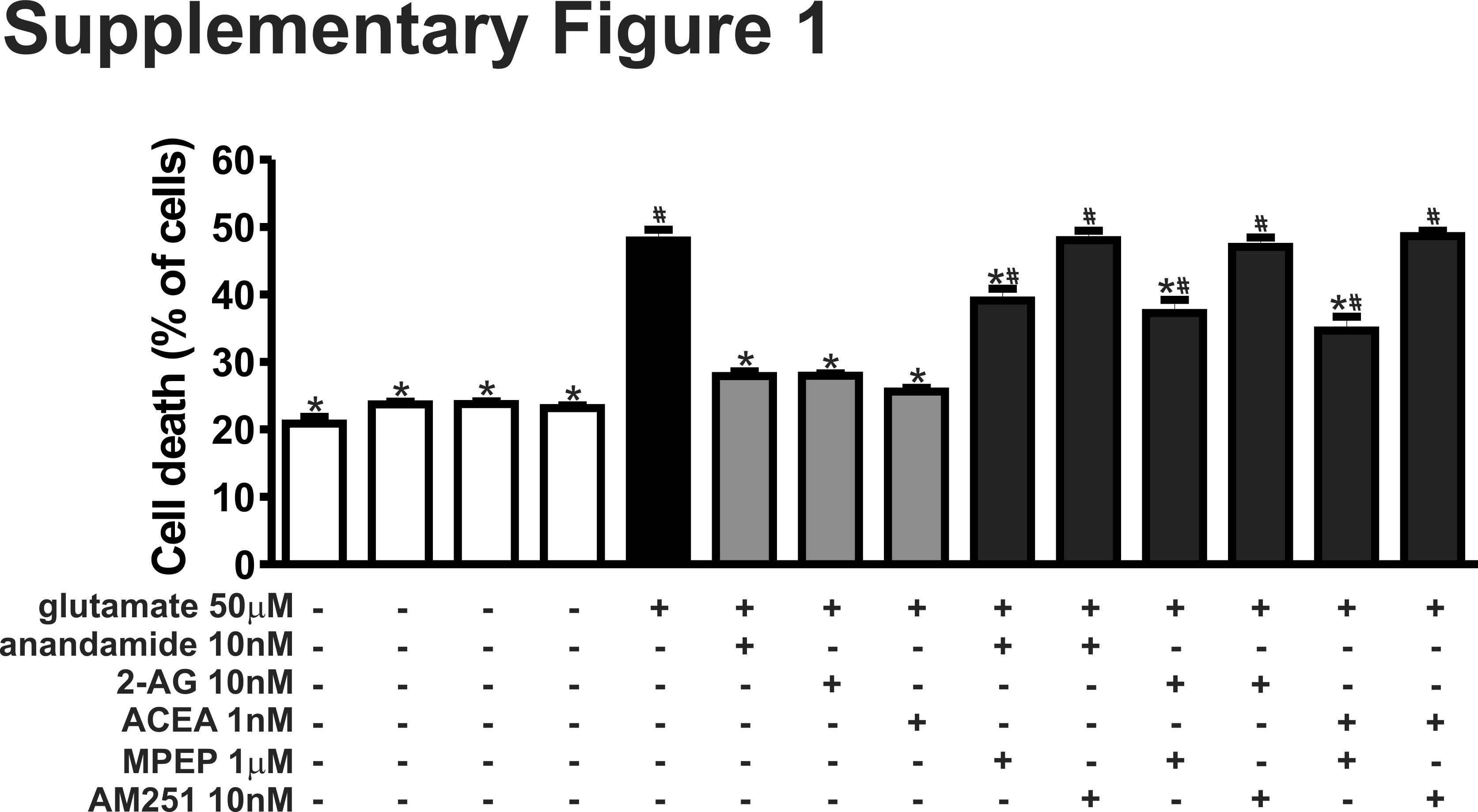

Supplement: Additional file 1: Figure S1. — Cannabinoid receptors direct agonists can promote neuroprotection in an mGluR5-independent manner. Graph shows cell death levels of primary cultured corticostriatal neurons that were either untreated (−) or treated (+) with 50 μM glutamate, 10 nM anandamide, 10 nM 2-AG, 1 nM ACEA, 1 μM MPEP and 10 nM AM251 for 4 h. Data represent the means ± SEM of four independent experiments. * indicates significant difference as compared to glutamate treated neurons and # indicates significant difference as compared to untreated neurons (p <0.05). (TIF 270 kb) [file 13041_2016_259_MOESM1_ESM.tif]

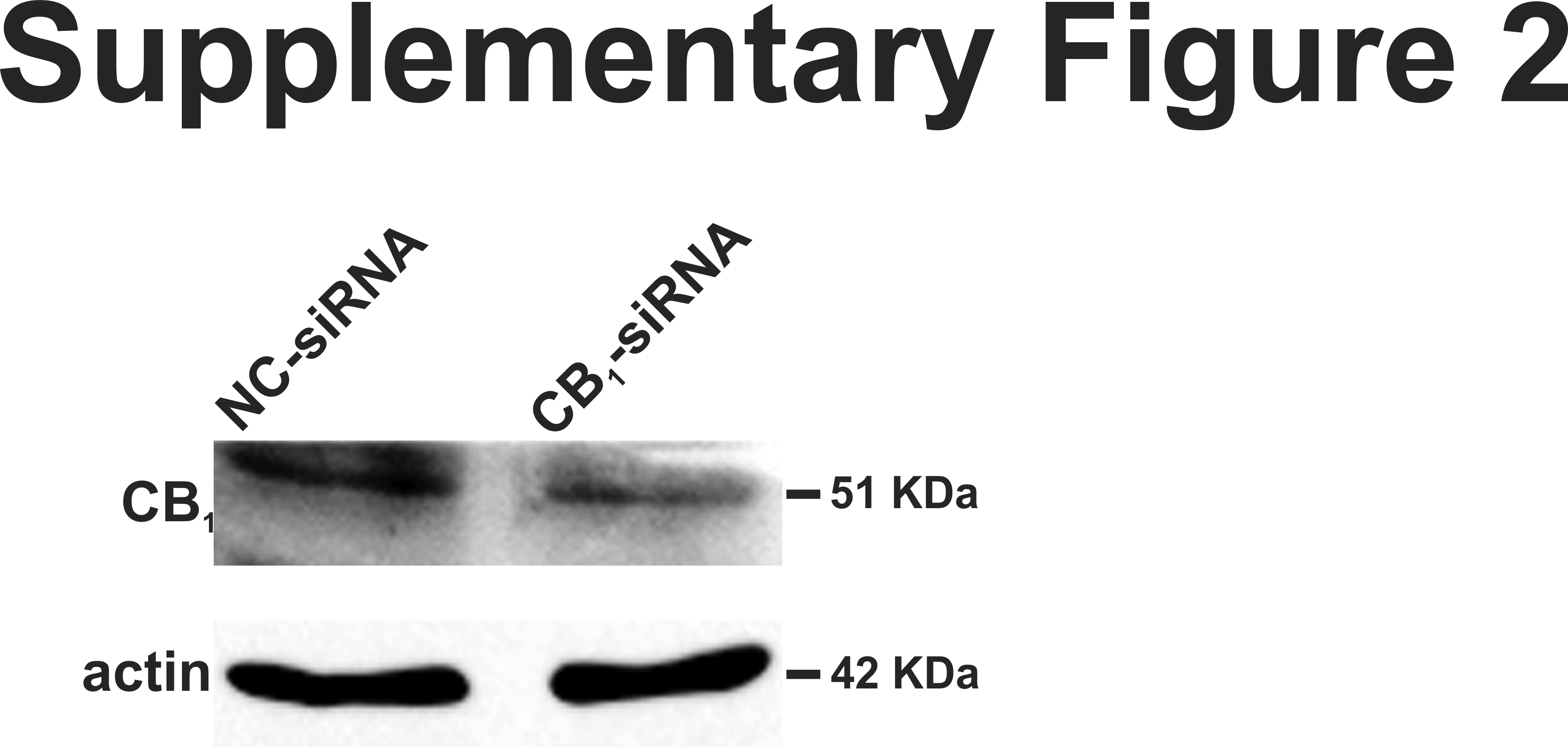

Supplement: Additional file 2: Figure S2. — CB1 siRNA electroporation decreases CB1 protein expression. Shown are representative immunoblots for CB1 (upper panel) and actin expression (lower panel) in primary cultured corticostriatal neurons that were electroporated with either NC- or CB1-siRNA. Immunoblots are representative of four independent experiments. (TIF 292 kb) [file 13041_2016_259_MOESM2_ESM.tif]

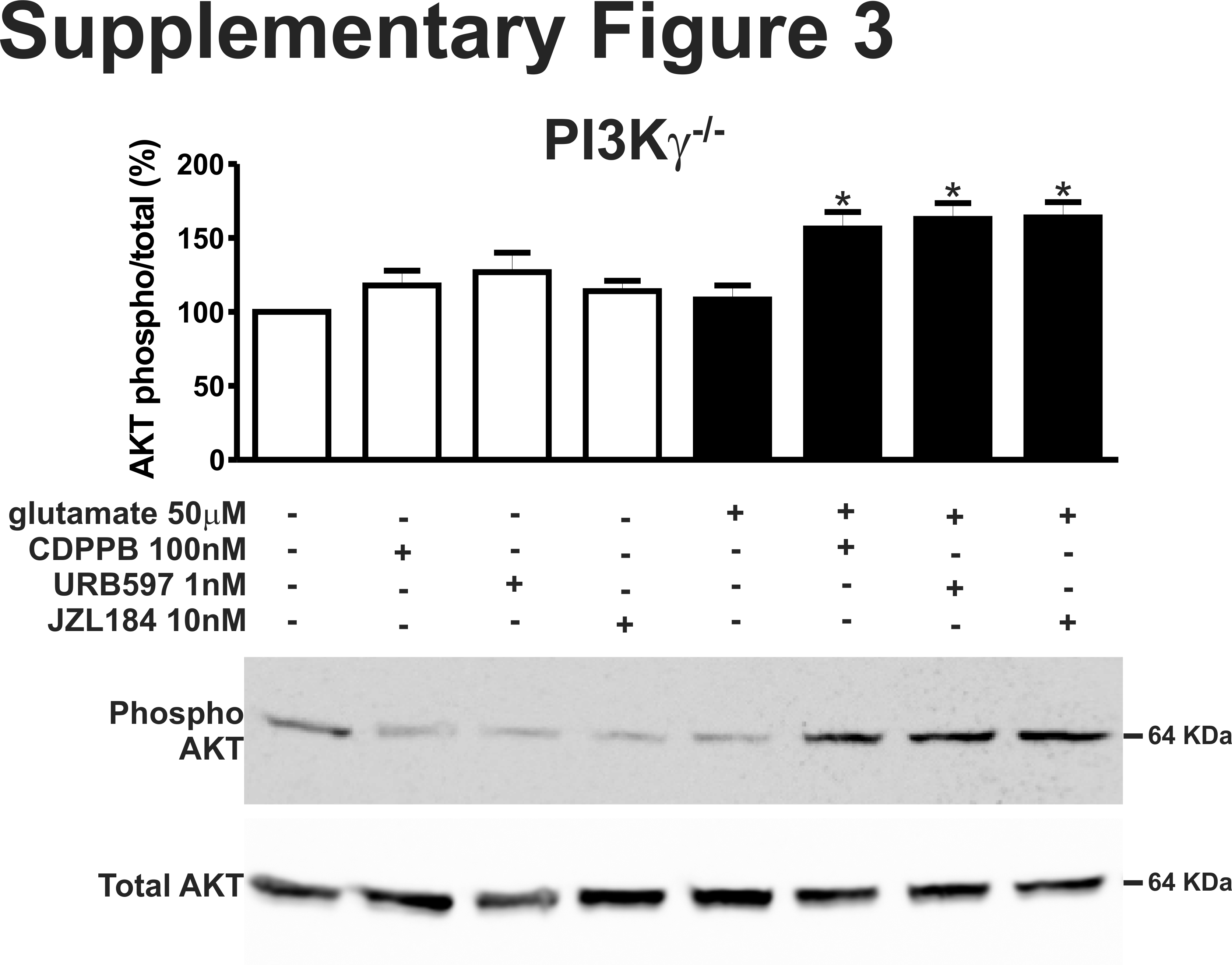

Supplement: Additional file 3: Figure S3. — AKT activation is decreased, but not abolished, in PI3Kγ−/− neurons. Shown are representative immunoblots for phospho- (upper panel) and total-AKT expression (lower panel) and graphs depicting the densitometric analysis of phospho-AKT normalized to total-AKT expression in primary cultured corticostriatal neurons from PI3Kγ−/− embryos that were either untreated (−) or treated (+) with 50 μM glutamate, 100 nM CDPPB, 1 nM URB597 and 10 nM JZL184 for 7.5 min. 100 μg of cell lysate was used for each sample. Data represent the means ± SEM of four independent experiments. * indicates significant difference as compared to untreated neurons (p <0.05). (TIF 568 kb) [file 13041_2016_259_MOESM3_ESM.tif]

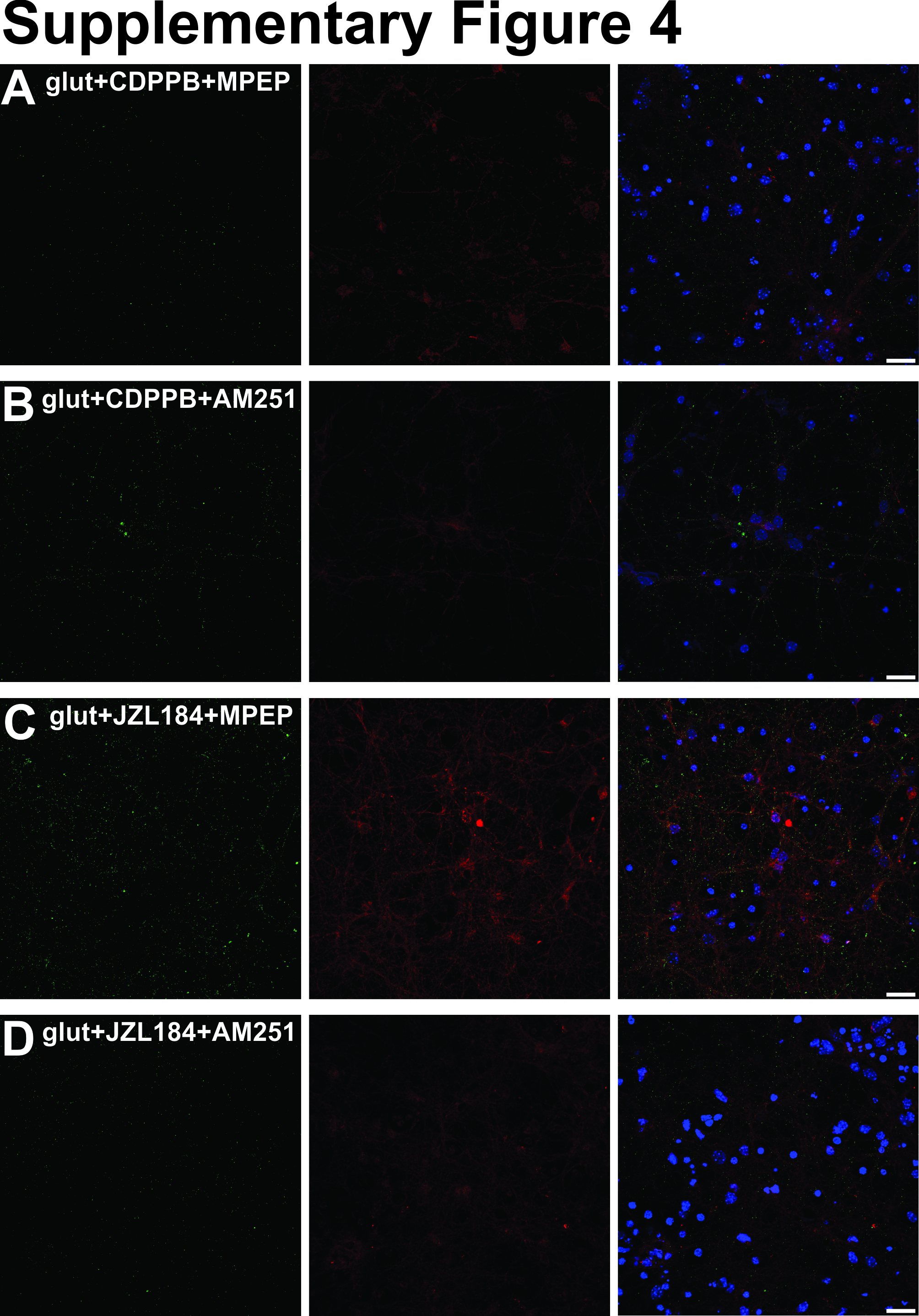

Supplement: Additional file 4: Figure S4. — MPEP and AM251 action on CDPPB- and JZL184-mediated protection of synaptic terminals. Shown are laser-scanning confocal micrographs depicting Alexa Fluor 488-conjugated anti-PSD95 antibody (green), Alexa Fluor 546-conjugated anti-syntaxin 1A antibody (red) and DAPI (blue) in neurons that were treated with 50 μM glutamate (glut) in the presence of 100 nM CDPPB + 1 μM MPEP (A), 100 nM CDPPB + 10 nM AM251 (B), 10 nM JZL184 + 1 μM MPEP (C) and 10 nM JZL184 + 10 nM AM251 (D) for 4 h. Scale bar = 20 μM. (TIF 8576 kb) [file 13041_2016_259_MOESM4_ESM.tif]
